# Integrative multi-omics profiling reveals coordinated immunometabolic reprogramming and host-microbiome interactions in acute pancreatitis

**DOI:** 10.3389/fimmu.2026.1828633

**Published:** 2026-06-19

**Authors:** Peng Dai, Jing Feng, Jianghong Cao, Daguang Fan

**Affiliations:** 1 Department of Hepato-Pancreatic-Biliary Surgery, Shanxi Provincial People’s Hospital, Taiyuan, China; 2 Department of Gastroenterology, Shanxi Provincial People’s Hospital, Taiyuan, China; 3 Department of Medical Intensive Care Unit, Shanxi Provincial People’s Hospital, Taiyuan, China

**Keywords:** acute pancreatitis (AP), candidate biomarkers, gut microbiome dysbiosis, immune-related gene expression, multi-omics integration

## Abstract

**Background:**

Acute pancreatitis (AP) is a life-threatening inflammatory disorder characterized by diverse etiologies and complex pathophysiological mechanisms involving immune dysregulation, systemic metabolic reprogramming, and gut microbiota disturbances. Although single-omics studies have provided partial insights into AP pathogenesis, comprehensive integrative multi-omics analyses investigating the intricate interactions among immunity, metabolism, and the microbiome in AP remain limited.

**Methods:**

We conducted an integrative multi-omics analysis of peripheral blood transcriptomics, untargeted plasma metabolomics, and fecal whole-metagenome sequencing in 15 patients with AP and 15 age- and sex-matched healthy controls. Differentially expressed genes (DEGs), metabolites (DEMs), and gut microbial species (DGMs) were identified. Subsequently, functional enrichment analysis, correlation network analysis, and exploratory machine learning approaches were employed to investigate molecular interactions and identify candidate biomarkers.

**Results:**

Transcriptomic profiling identified 4, 776 DEGs, including 409 immune-related genes significantly enriched in the NF-κB, IL-17, and cytokine-cytokine receptor interaction pathways, indicating pronounced inflammatory activation. Metabolomic analysis detected 296 DEMs, with prominent alterations in amino acid and lipid metabolism, mong which 9 metabolites showed potential discriminatory value (AUC > 0.75), with representative metabolites including xanthine, homocarnosine, and tetradecanedioic acid. Metagenomic sequencing revealed significant microbial compositional and functional remodeling, characterized by enrichment of pro-inflammatory taxa such as *Escherichia coli* and *Streptococcus anginosus*, alongside depletion of SCFA-producing commensals including *Faecalibacterium prausnitzii* and *Blautia wexlerae*. Functional profiling demonstrated disrupted amino acid metabolism, gut-brain signaling, and SCFA synthesis. Multi-omics integration revealed 215 significant correlations between host genes, metabolites, and microbes, highlighting key interaction hubs. An exploratory random forest model identified *Lachnospira pectinoschiza*, *Megamonas funiformis*, and SRGN as candidate biomarkers, showing promising classification performance within the current cohort (AUC = 0.951).

**Conclusions:**

This study provides a systems-level characterization of the immune, metabolic, and microbial alterations in AP. The identified molecular signatures and cross-omics interaction networks offer mechanistic insights into AP pathogenesis and highlight candidate biomarkers that warrant further validation in larger, independent cohorts.

## 1 Introduction

Acute pancreatitis (AP) is an inflammatory condition of the pancreas characterized by self-digestion of pancreatic tissue, which leads to a clinical spectrum ranging from mild, self-limiting episodes to severe, life‐threatening conditions accompanied by systemic inflammatory response syndrome and multiple organ dysfunction (1, 2). The global incidence of AP is rising, with approximately 34 cases per 100, 000 individuals annually, and the mortality rate in severe cases remains high, ranging from 10% to 30% (3–5).

Recent advances in high-throughput multi-omics technologies have provided unprecedented opportunities to decode the complex pathophysiological mechanisms underlying AP. Transcriptomic analyses of pancreatic tissue and peripheral blood mononuclear cells have uncovered the activation of NF-κB-mediated inflammatory pathways, oxidative stress responses, and apoptosis pathways in necrotizing AP (6). Single-cell RNA sequencing has further delineated the dynamic immune cell infiltration and heterogeneity during AP, highlighting increased macrophage and neutrophage abundance, aberrant cell-cell communication, and disease-specific gene signatures such as Lcn2, Cd44, and Osmr (7). Complementarily, metabolomics studies have identified significant alterations in lipid, amino acid, and energy metabolism that reflect both localized pancreatic injury and systemic metabolic reprogramming during AP progression (8, 9). Mounting evidence underscores the crucial role of the gut microbiome in AP pathogenesis. Dysbiosis, characterized by a depletion of short-chain fatty acid (SCFA)-producing commensals (e.g., Faecalibacterium prausnitzii) and an overgrowth of opportunistic pathobionts (e.g., Enterobacteriaceae), has been linked to impaired gut barrier function, bacterial translocation, and systemic endotoxemia (10–13). Large-scale clinical cohort studies have demonstrated that microbiota composition and function can stratify disease severity and predict outcomes more accurately than conventional scoring systems (7). Consequently, these microbial features are being explored not only as diagnostic markers but also as immunomodulatory targets due to their roles in maintaining epithelial integrity and tempering inflammation (14).

On the immunological level, AP is recognized as a biphasic disorder characterized by an initial excessive activation of innate immune cells (e.g., neutrophils and monocytes) and inflammatory mediators (e.g., TNF-α and IL-1β), followed by an anti-inflammatory or immunosuppressive phase marked by T cell exhaustion, regulatory T cell dysregulation, and increased susceptibility to infection (15, 16). Recent multi-omics investigations have revealed significant disruptions in immune checkpoints, such as the PD-1/PD-L1 axis, and shifts in the balance between innate and adaptive immune compartments, particularly during the early stages of AP (17). Moreover, emerging studies have begun to identify specific immune-metabolic and microbiota-host interaction hubs-including microbial metabolites like SCFAs and immune-regulatory genes such as PD-L1 and LY96-that are closely associated with AP progression and severity (18, 19).

However, despite these advances, critical gaps impede the translation of multi omics discoveries into clinical practice. While gallstones and alcohol account for the majority of AP cases, the etiopathogenesis and mechanistic underpinnings of less common causes, such as hypertriglyceridemia, drug induced injury, and genetic predisposition remain insufficiently characterized (20). Early stratification of disease severity at the time of admission continues to rely on conventional biomarkers (e.g., CRP, interleukin-6) and clinical scoring systems (e.g., Ranson, BISAP), yet these tools achieve only moderate predictive accuracy (~75%), leading to delays in risk-adapted management and poorer outcomes (21). Although numerous candidate biomarkers, such as phospholipase D2 (PLD2) transcripts and specific lipidomic signatures, have been identified in small, single-center cohorts, they lack robust, multicenter validation necessary for broad clinical adoption. Furthermore, no omics-derived therapeutic targets have progressed to clinical trial evaluation, underscoring a translational gulf between mechanistic insights and therapeutic innovation (22). Furthermore, the majority of published studiesemploy cross sectional designs and focus on single omics layers, which limits a comprehensive understanding of the intricate crossstalk between host, microbiota, and metabolism in AP. To overcome these limitations, integrative multi-omics approaches combining transcriptomics, metabolomics, and metagenomics offer a holistic framework to unravel the complex crosstalk between host immune responses, metabolic alterations, and microbial ecosystems in AP. These systems-level strategies have proven successful in other inflammatory and metabolic diseases and hold promise for uncovering novel biomarkers and therapeutic targets that would be missed by single-omics analyses alone (23, 24). Recent advances in multi-omics research have demonstrated that integrating datasets across transcriptional, metabolic, and genomic layers can uncover clinically relevant molecular subtypes and key regulatory pathways that are often difficult to detect using single-omics approaches. This integrative analytical strategy has proven particularly powerful for characterizing disease heterogeneity and identifying cross-level interactions among immune signaling, metabolic remodeling, and cellular stress regulation (25). Given that acute pancreatitis involves tightly interconnected immune activation, metabolic dysfunction, and gut microbiota imbalance, applying a multi-omics framework is expected to offer a more comprehensive and holistic understanding of its underlying pathophysiology.

Therefore, the present study employs an integrative multi-omics approach-combining transcriptomics, metabolomics, and metagenomics-to systematically characterize host-microbiota-metabolite interaction networks in AP. By elucidating these complex crosstalk mechanisms, we aim to identify potential mechanistic hubs and candidate diagnostic biomarkers with translational potential.

## 2 Materials and methods

### 2.1 Study population

This study enrolled 15 patients with acute pancreatitis (AP) and 15 age-and sex-matched healthy controls (HCs) from Shanxi Provincial People’s Hospital (Taiyuan, China). The diagnosis of AP was based on the revised Atlanta classification criteria, with all patients showing elevated levels of total bilirubin, liver enzymes, glucose, and triglycerides. Individuals with a history of gastrointestinal diseases, malignancy, or immune disorders were excluded. For all enrolled AP patients, detailed clinical data including etiology, history of alcohol consumption and smoking, and underlying comorbidities (such as hypertension, diabetes mellitus, and hyperlipidemia) were collected through electronic medical records and patient interviews. To minimize confounding effects on the gut microbiota, patients who had recently received antibiotics or were unable to provide fecal samples within 48 hours of admission were not included.

Fecal samples were collected under standardized conditions using sterile containers and transported on ice to the laboratory within 30 minutes. Upon arrival, each sample was aliquoted into multiple tubes and stored at −80 °C until further processing. The study protocol was approved by the Ethics Committee of Shanxi Provincial People’s Hospital (approval number: SXSPH-2021--EC-018), and written informed consent was obtained from all participants or their legal guardians.

### 2.2 Transcriptome sequencing and analysis

Peripheral blood (2–3 mL) was collected from fasting subjects into EDTA tubes. Peripheral blood mononuclear cells (PBMCs) were isolated by density gradient centrifugation and used for RNA extraction. Total RNA was extracted using TRIzol reagent (Invitrogen, USA) according to the manufacturer’s instructions, RNA samples were treated with RNase-free DNase I to remove potential genomic DNA contamination. RNA integrity was assessed using the Agilent 2100 Bioanalyzer (Agilent Technologies, USA), and RNA concentration was measured using a Qubit 4.0 Fluorometer. Only samples with RNA integrity number (RIN) ≥ 7.0 were used for downstream analysis.

RNA-seq libraries were constructed using the NEBNext^®^ Ultra™ Directional RNA Library Prep Kit for Illumina following the manufacturer’s protocol. Poly(A) mRNA was enriched using oligo(dT) beads prior to library preparation. The libraries were sequenced on an Illumina HiSeq 2000 platform (Personalbio Biotechnology, Shanghai, China). Raw reads were processed using Trimmomatic (v0.33) to remove adapters and low-quality sequences, Reads with more than 10% ambiguous bases or with quality scores < 20 were discarded. Clean reads were aligned to the human reference genome (GRCh38) using HISAT2. Gene expression was quantified using HTSeq (v0.7.2) based on aligned reads to generate raw count data. Differential expression analysis was performed using DESeq2 (v1.18.0), which utilizes raw counts and applies its own normalization method to identify DEGs. Genes with |log2FC| ≥ 1 and adjusted P-value < 0.05 were considered significantly differentially expressed. FPKM values were calculated based on gene length and sequencing depth and were used for visualization and expression comparison across samples. Principal component analysis (PCA) was performed using the prcomp function in R to assess sample clustering based on gene expression profiles. Pearson correlation coefficients between samples were calculated to evaluate data consistency and reproducibility, and visualized using the pheatmap package. Functional annotation of DEGs was performed using Gene Ontology (GO) and Kyoto Encyclopedia of Genes and Genomes (KEGG) enrichment analyses with the R packages clusterProfiler (v4.0) and ggplot2. In addition, gene set enrichment analysis (GSEA) was conducted using the fgsea package (v1.20.0) to identify significantly enriched biological pathways (adjusted P-value < 0.05).

### 2.3 Untargeted metabolomic sequencing and analysis

Plasma was isolated from peripheral venous blood by centrifugation at 3, 000 rpm at 4 °C for 10 min. Subsequently, 100 μL of plasma was transferred into an EP tube and mixed with 400 μL of methanol containing chlorphenamine maleate as an internal standard. After vortexing and incubation at −40 °C for 1 h, samples were centrifuged again at 3, 000 rpm for 10 min, and 200 μL of the supernatant was collected for metabolomic analysis. A pooled quality control (QC) sample was prepared by combining aliquots from all individual samples to monitor analytical stability.

Metabolomic profiling was performed using an AB SCIEX QTRAP 6500+ system (AB SCIEX, USA) coupled with a Shimadzu Nexera X2 UHPLC system. Chromatographic separation was conducted on a Thermo Scientific Hypersil GOLD C18 column (2.1 mm × 100 mm, 1.7 μm) maintained at 40 °C. The mobile phase consisted of solvent A (0.1% formic acid in water) and solvent B (0.1% formic acid in acetonitrile), delivered at a flow rate of 0.3 mL/min. The gradient elution program was as follows: 0–2 min, 98% A; 2–10 min, linear gradient to 5% A; 10–12 min, 5% A; and 12–15 min, re-equilibration to 98% A. Mass spectrometry data were acquired in both positive and negative electrospray ionization modes. The ion source parameters were set as follows: ion spray voltage, +5500 V/-4500 V; source temperature, 500 °C; ion source gas 1 and gas 2, 55 psi; curtain gas, 35 psi; and declustering potential, ± 80 V. Data were acquired in full MS scan mode (m/z 100–1000) and information-dependent acquisition (IDA) mode for MS/MS analysis.

Raw data were processed using Analyst software (v1.6.3, AB SCIEX) for peak detection, alignment, and integration. Metabolite identification was performed by matching exact mass values and MS/MS fragmentation spectra against the Human Metabolome Database (HMDB) and METLIN database. Annotation confidence was assigned according to Metabolomics Standards Initiative criteria. Multivariate statistical analyses were performed using SIMCA-P+ (v15.0, Sartorius, Sweden). Principal component analysis (PCA) was first applied for unsupervised overview and outlier detection, followed by orthogonal partial least squares-discriminant analysis (OPLS-DA) to maximize group separation. Model robustness was evaluated using permutation testing and cross-validated R²Y/Q² metrics. Metabolites with a variable importance in projection (VIP) score ≥1.0, |fold change| ≥ 2, and P < 0.05 (two-tailed Student’s t-test) were considered differentially abundant metabolites. Pathway enrichment analysis was performed using MetaboAnalyst (v5.0) to map differential metabolites to KEGG pathways. Receiver operating characteristic (ROC) analysis was subsequently performed to assess the discriminatory potential of selected metabolites, and area under the curve (AUC) values were reported.

### 2.4 Whole-metagenome shotgun sequencing and analyzing of gut microbiome

To characterize gut microbial composition and function, whole-metagenome shotgun sequencing was performed. Fecal samples (≥500 mg) were collected using sterile scoops, immediately frozen at −80 °C, and subsequently used for microbial DNA extraction with the QIAamp DNA Stool Mini Kit (Qiagen, Germany). DNA quality and concentration were assessed using a Qubit fluorometer and agarose gel electrophoresis. DNA libraries were constructed from randomly fragmented DNA and sequenced using 150-bp paired-end reads on the MGI-SEQ2000 platform (MGI, Shenzhen, China).

Raw data were filtered using SOAPnuke v1.5.2 (26) and KneadData v0.10.0 to remove low-quality reads, adapter sequences, and host-derived contamination. Taxonomic profiling was performed using MetaPhlAn3 for species-level quantification based on clade-specific marker genes. To enable broader detection of non-bacterial microorganisms, including archaea, viruses, and fungi, additional taxonomic classification was conducted using Kraken2 (v2.1.2) with a customized reference database derived from NCBI RefSeq, FungiDB, and Ensembl Fungi. Taxonomic abundance estimates were further refined using Bracken (v2.5.0). Functional profiling was performed using HUMAnN3 (v3.9) through the bioBakery pipeline (27), which quantified microbial gene families and reconstructed MetaCyc pathways and KEGG ortholog (KO) abundance profiles. Comparative analysis of microbial taxa between groups was conducted using MaAsLin2 with adjustment for age, sex, and BMI. Taxa with adjusted Q-values < 0.05 were considered significantly differentially abundant. LEfSe analysis was subsequently performed as a complementary exploratory approach to identify taxonomic enrichment patterns. To further prioritize microbial features associated with AP, a random forest model was applied to differentially abundant microbial species using the R package randomForest (v4.7-1.1) with parameters set as ntree = 500 and mtry = 7. Feature importance was ranked based on Mean Decrease Accuracy (MDA), and top-ranked taxa were retained as candidate microbial biomarkers for downstream analysis. Spearman correlation analysis was performed to evaluate associations between microbial species abundance and host-related functional features in AP, including clinical parameters, gut metabolite modules (GBMs), gut-brain modules (GMMs), KEGG pathways, and KEGG orthologs (KOs). P-values were adjusted using the Benjamini-Hochberg false discovery rate (FDR) method, and an adjusted P < 0.05 was considered statistically significant.

### 2.5 Construction of multi-omics correlation network

To investigate the interactions among host immunity, metabolism, and gut microbiota, Spearman correlation analysis was performed to assess pairwise associations among differentially expressed genes (DEGs), differentially expressed metabolites (DEMs), and differentially abundant microbial taxa (DGMs). Significant correlations were defined as an absolute Spearman correlation coefficient > 0.6 and a false discovery rate (FDR)-adjusted P-value < 0.05. Subsequently, multiple linear regression analysis was performed using immune-related DEGs as dependent variables and DEMs and DGMs as independent variables to identify key microbial and metabolic predictors of host immune responses. This integrative strategy was used to characterize disease-associated molecular interaction networks in AP. To further identify candidate biomarkers, a random forest (RF) model was constructed based on significantly associated features derived from the multi-omics correlation network. RF analysis was performed using the R package randomForest (v4.7-1.1) (ntree = 500, mtry = 7). Recursive feature selection was conducted using the rfcv function with repeated 10-fold cross-validation to identify the optimal biomarker combination. Feature importance was ranked based on Mean Decrease Accuracy (MDA), and model performance was evaluated using receiver operating characteristic (ROC) analysis and area under the curve (AUC) values calculated with the pROC package. Due to the limited sample size, no independent validation cohort was available; therefore, the reported AUC reflects internal model performance and should be considered exploratory.

### 2.6 Expression validation

To further verify the expression patterns of key differentially expressed genes, eight genes (FTL, F13A1, CEBPD, SRGN, RGS1, LGALS1, FN1, and EGR1) were selected based on their top-ranking importance scores in the random forest model. Both mRNA and protein expression levels were evaluated. For transcript-level validation, gene-specific primers were designed using Premier 5 and confirmed for specificity via NCBI BLAST. Primer sequences are provided in **Table 1**. qRT-PCR was performed on an ABI ViiA 7 system using SYBR Green Master Mix (TaKaRa, Japan) in a 15 μL reaction containing 2.0 μL cDNA, 7.5 μL 2x mix, 1.5 μL primers (2.5 μM), and 4.0 μL nuclease-free water. GAPDH was used as the internal control. All reactions were run in triplicate, and relative expression levels were calculated using the 2 ^-△△CT^ method.

**Table 1:** Primer sequence.

Genes	Forward sequence (5′→3′)	Reverse sequence (5′→3′)
FTL	AGAACTTGGACAGCAGCAGG	GAGCAGCTCCTCCTCATAGC
F13A1	TGAAGGCTGCTGAGGAAGAAG	TGGCAGGTGTAGCTGAGTGA
CEBPD	CAGCCTTCTTGGAAGTGGTG	GTTGGGCTGGAAGTAGTCGA
SRGN	AGGAGGAGGAAGGAGGAGGA	TGCAGGTTGGGTTTGATGTT
RGS1	CTCTGCTTCGAGGAAGACGA	AGAGATGGTGGAGGGAAGGA
LGALS1	CCTTTGGCTCATGTGCTGGAAC	GCCATAAGTGTGGGTTTCAGTAC
FN1	GCCTGGTACAGAATATGTAGTG	ATCCCAGCTGATCAGTAGGCTGGTG
EGR1	TGAGGTGGTGATGCTGGTTC	TGGGTTGTGTCATAGCCAGA
GAPDH	TGTGTCCGTCGTGGATCTGA	TTGCTGTTGAAGTCGCAGGAG

For protein-level validation, total protein was extracted using 1× SDS lysis buffer and quantified with a BCA protein assay kit (Pierce, USA). Equal amounts of protein were separated by 15% SDS-PAGE and transferred onto PVDF membranes (Millipore, USA). After blocking with 5% non-fat milk, membranes were incubated overnight at 4 °C with primary antibodies. Detailed information for all primary and secondary antibodies, including manufacturers, catalog numbers, and dilution ratios, is provided in **Supplementary Table 1**. After washing, membranes were incubated with appropriate HRP-conjugated secondary antibodies for 2 h at room temperature. Protein bands were visualized using chemiluminescence and normalized to GAPDH.

### 2.7 Statistical analysis

Statistical analyses were performed using SPSS (version 26) and the R software (version 4.0). Comparisons between two groups were calculated by Student’s t test. In addition, Spearman correlation analysis was applied to assess associations based on relative abundance data. To account for multiple comparisons, p-values were adjusted using the Benjamini-Hochberg false discovery rate (FDR) method. An adjusted P < 0.05 was considered statistically significant.

## 3 Results

### 3.1 Clinical characteristics of AP patients

A total of 30 participants were enrolled in this study, including 15 patients with AP and 15 healthy controls (HCs). Detailed baseline characteristics are summarized in **Table 2**. The two groups were well matched in terms of age, sex, and body mass index (BMI) (all P > 0.05). Among patients with AP, biliary stones were the most common etiology (60.0%), followed by hypertriglyceridemia (26.7%). According to the revised Atlanta classification, most cases (73.3%) were classified as mild AP. No significant differences were observed between the AP and HC groups in smoking status, alcohol consumption, or the prevalence of major comorbidities, including hypertension, diabetes mellitus, and hyperlipidemia (all P > 0.05), indicating good baseline comparability between groups.

**Table 2:** Clinical characteristics of AP patients.

Characteristic	AP N = 15	HC N = 15	P-value
Demographics			
Gender (F/M)	9/6	9/6	>0.99911
Age, year	59.07 ± 5.89	60.20 ± 7.10	0.63822
Height (cm)	161.67 ± 6.86	162.53 ± 6.81	0.73122
Weight (kg)	61.36 ± 7.59	60.20 ± 5.43	0.63422
Body mass index, BMI (kg/m2)	23.46 ± 2.42	22.87 ± 2.55	0.52022
Etiology of AP, n(%)			
Biliary	9 (60.0%)	–	–
Hypertriglyceridemia (HTG)	4 (26.7%)	–	–
Alcoholic	1 (6.7%)	–	–
Other/Idiopathic	1 (6.7%)	–	–
Disease Severity (ATS, n(%))			
Mild	11 (73.3%)	–	–
Moderately Severe	3 (20.0%)	–	–
Severe	1 (6.7%)	–	–
Lifestyle Factors			
Current Smoker	5 (33.3%)	4 (26.7%)	0.695 1
Regular Alcohol Use	4 (26.7%)	3 (20.0%)	0.676 1
Comorbidities			
Hypertension	6 (40.0%)	5 (33.3%)	0.714 1
Diabetes Mellitus	3 (20.0%)	2 (13.3%)	0.630 1
Hyperlipidemia	7 (46.7%)	4 (26.7%)	0.2631
Laboratory Parameters			
White blood cell, WBC (10^9/L)	15.50 ± 4.17	5.93 ± 1.18	<0.00122
% Neutrophil cell, %NEUT	83.60 ± 6.09	60.30 ± 5.06	<0.00122
Alanine transaminase, ALT (U/L)	164.67 ± 80.59	14.33 ± 3.54	<0.00122
Aspartate aminotransferase, AST (U/L)	137.21 ± 96.89	15.37 ± 2.21	<0.00122
γ-Glutamyl transpeptidase, γ-GT (U/L)	215.78 ± 116.91	22.55 ± 11.64	<0.00122
Alkaline phosphatase, ALP (U/L)	157.02 ± 69.78	87.80 ± 41.21	0.00322
Total bilirubin, TB (μmol/L)	52.60 ± 23.21	9.95 ± 1.64	<0.00122
Direct bilirubin, DB (μmol/L)	27.89 ± 8.20	1.65 ± 0.53	<0.00122
Total cholesterol, CHOL (mmol/L)	6.02 ± 2.80	4.30 ± 0.36	0.03322
Triglycerides, TG (mmol/L)	5.09 ± 1.72	1.01 ± 0.22	<0.00122
Fasting glucose, Glu (mmol/L)	8.27 ± 1.78	5.26 ± 0.49	<0.00122
Urea (mmol/L)	5.87 ± 1.41	4.91 ± 0.71	0.02922

1 Pearson’s Chi-squared test; ^2^Welch Two Sample t-test; The data are presented as the mean ± SD. n.d., no data. P-values are calculated based on the Fisher’s exact test (gender) and Student’s t-test (other parameters). -: Not applicable.

However, significant differences were observed in several hematological and biochemical parameters. Compared with HCs, patients with AP exhibited significantly elevated levels of white blood cells (WBC), neutrophil percentage (%NEUT), alanine transaminase (ALT), aspartate aminotransferase (AST), γ-glutamyl transpeptidase (γ-GT), alkaline phosphatase (ALP), total bilirubin (TB), direct bilirubin (DB), total cholesterol (CHOL), triglycerides (TG), fasting glucose (Glu), and urea (all P < 0.05). These findings reflect the systemic inflammatory and metabolic disturbances present in AP and provide important clinical context for subsequent multi-omics analyses.

### 3.2 Transcriptome sequencing results

**Figure 1:**
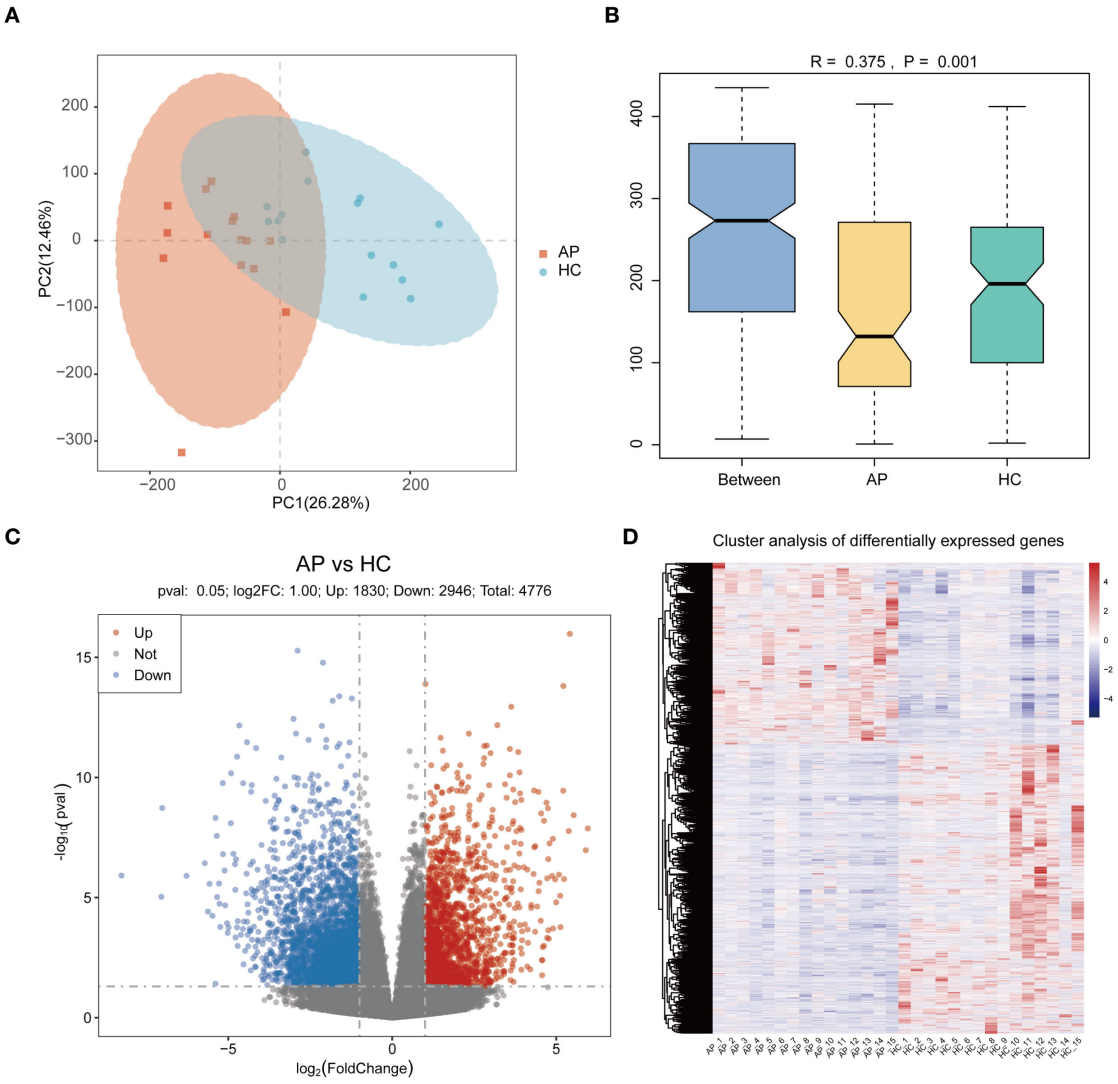
Transcriptomic profiling reveals distinct gene expression signatures in AP: **(A)** Principal component analysis (PCA) based on normalized gene expression profiles. Each point represents one sample, and the distances between points reflect overall transcriptomic differences among samples. **(B)** ANOSIM analysis confirms significant differences between groups (R > 0), with boxplots showing between- and within-group dissimilarities. **(C)** Volcano plot of differentially expressed genes (DEGs); red and blue dots represent significantly upregulated and downregulated genes in AP, respectively (|log2FC| >1, P < 0.05). **(D)** Heatmap of DEGs expression across samples; each row represents a gene and each column a sample. Red indicates high expression, blue indicates low.

High-throughput sequencing generated 1, 361, 873, 520 raw reads (204.16 Gb), yielding 1, 341, 525, 278 high-quality clean reads (201.23 Gb; Q20 = 97.70%; GC content = 48.15%) after filtering. More than 95% of clean reads were successfully mapped to the human reference genome, indicating high data quality and mapping efficiency (**Supplementary Table 1**). Principal component analysis (PCA) demonstrated clear separation between AP and HC groups, with PC1 and PC2 explaining 26.28% and 12.46% of the variance, respectively (**Figure 1A**). This separation was further supported by ANOSIM analysis (R = 0.375, P = 0.001; **Figure 1B**). According to the predefined criteria, a total of 4, 776 differentially expressed genes (DEGs) were identified, including 1, 830 upregulated genes and 2, 946 downregulated genes (**Figure 1C**, **Supplementary Table 2**). A heatmap of DEG expression patterns further demonstrated a predominance of downregulated transcripts in the AP group compared with HCs (**Figure 1D**).To investigate the biological significance of these DEGs, Gene Ontology (GO), Kyoto Encyclopedia of Genes and Genomes (KEGG) pathway analysis, and gene set enrichment analysis (GSEA) were performed.

**Figure 2:**
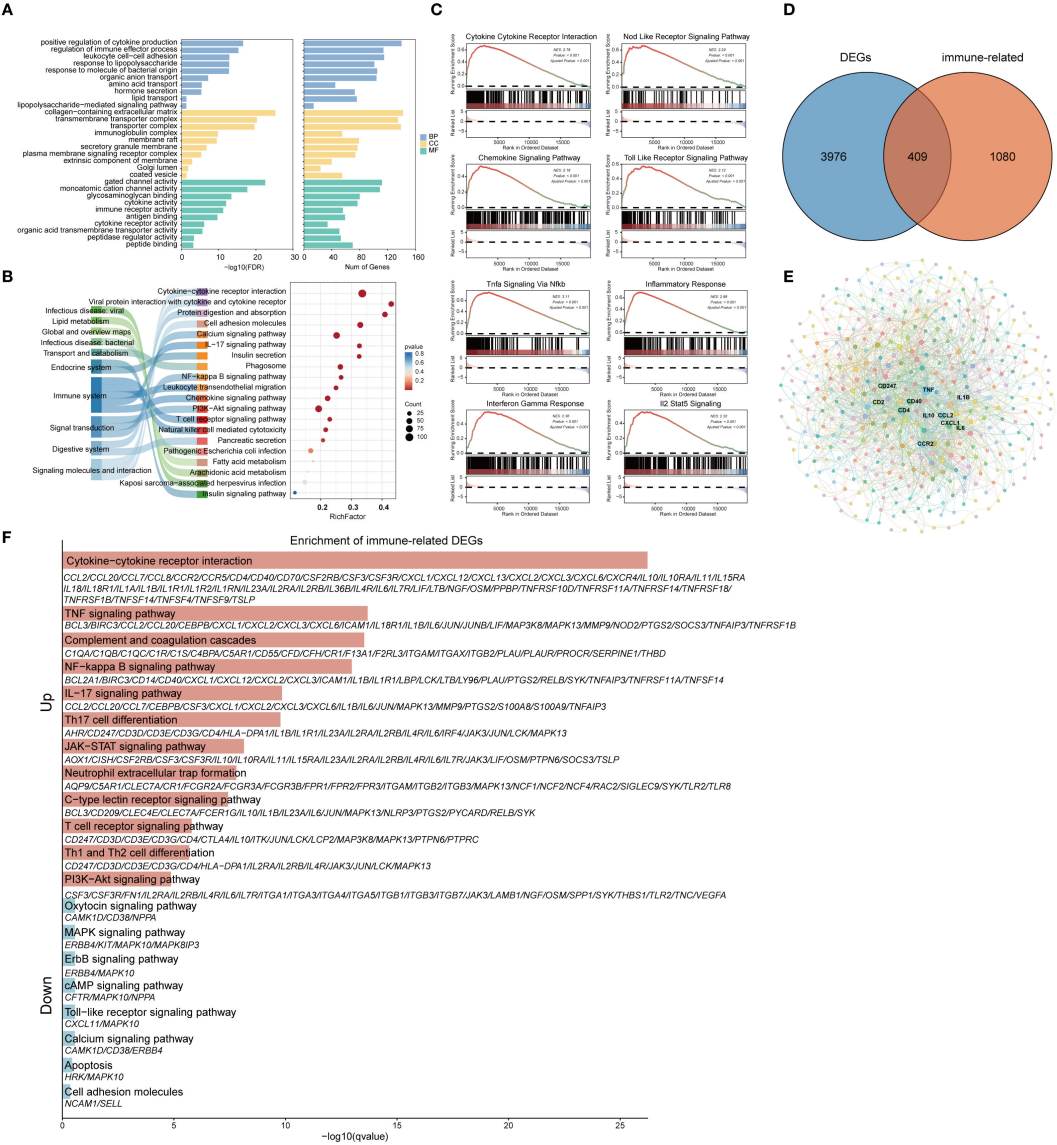
Functional enrichment and immune network analysis of DEGs in AP: **(A, B)** Gene Ontology (GO) and Kyoto Encyclopedia of Genes and Genomes (KEGG) Pathway Enrichment Analysis of DEGs. **(C)** GSEA analysis showed key biological processes and pathways involved in DEGs. **(D)** Venn diagram shows 409 genes that were common to DEGs and the immune-related genes in DB databases. **(E)** PPI network constructed based on immune-related DEGs. the dots represent immune-related DEGs. The larger size of the dots indicates more interactions with other genes in the network. **(F)** Functional enrichment analysis of immune-related DEGs. Red represent upregulated DEGs, while blue represent downregulated genes.

GO enrichment analysis revealed significant enrichment across biological process (BP), molecular function (MF), and cellular component (CC) categories, with BP terms showing the most prominent enrichment. The top 30 enriched GO terms are presented in **Figure 2A**. The most significantly enriched BP terms included positive regulation of cytokine production, regulation of immune effector processes, and leukocyte cell-cell adhesion. In the MF category, the top enriched terms were gated channel activity, monoatomic ion channel activity, and glycosaminoglycan binding. In the CC category, collagen-containing extracellular matrix, transmembrane transporter complex, and transporter complex were the most enriched terms. These findings suggest that transcriptional alterations in AP are primarily associated with immune activation, ion transport dysregulation, and extracellular matrix remodeling, which may contribute to inflammatory injury.

KEGG enrichment analysis revealed significant alterations in pathways related to the immune system, endocrine system, signal transduction, and digestive system (**Figure 2B**). Among the top 20 enriched pathways, significantly enriched pathways (P < 0.05) included leukocyte transendothelial migration, chemokine signaling pathway, T cell receptor signaling pathway, natural killer cell-mediated cytotoxicity, NF-κB signaling pathway, cell adhesion molecules, IL-17 signaling pathway, cytokine-cytokine receptor interaction, and protein digestion and absorption. These findings suggest that extensive activation of both innate and adaptive immune pathways may contribute to the systemic inflammatory response observed in AP. Gene set enrichment analysis (GSEA) further demonstrated significant upregulation of cytokine-cytokine receptor interaction, NOD-like receptor signaling pathway, TNFα signaling via NF-κB, inflammatory response, Toll-like receptor signaling pathway, chemokine signaling pathway, interferon-γ response, and IL2-STAT5 signaling in AP compared with HCs (**Figure 2C**). These results further support the presence of coordinated inflammatory signaling activation in AP. Given the prominent enrichment of immune-related pathways, we further intersected the identified DEGs with immune-related genes obtained from the relevant immune database to identify immune-associated DEGs. A total of 409 immune-related DEGs were ultimately identified (**Figure 2D**, **Supplementary Table 3**).

To investigate interactions among these immune-related DEGs, a high-confidence protein-protein interaction (PPI) network (interaction score > 0.9) was constructed using the the STRING database (**Figure 2E**). The network consisted of 407 nodes and 852 significant edges, with an average node degree of 4.19 and an average local clustering coefficient of 0.446. The number of observed edges was markedly higher than expected (852 vs. 96), indicating significant network interconnectivity (PPI enrichment P-value < 1.0e-16). Key hub genes, including IL6, IL1B, CXCL8, CD4, CD86, and TLR4, were located at the network core, suggesting their potential central roles in AP-associated immune dysregulation. To further characterize the biological functions of immune-related DEGs, pathway enrichment analysis was performed (**Figure 2F**). Upregulated genes were primarily enriched in immune and inflammatory pathways, including cytokine-cytokine receptor interaction, TNF signaling, IL-17 signaling, JAK-STAT signaling, NF-κB signaling, and T cell receptor signaling, reflecting robust pro-inflammatory activation in AP. In contrast, downregulated genes were mainly enriched in MAPK signaling, calcium signaling, cAMP signaling, ErbB signaling, Toll-like receptor signaling, and apoptosis-related pathways, suggesting suppression of specific signaling and immune regulatory processes. Collectively, these findings highlight the complex and imbalanced immune responses underlying AP pathophysiology.

### 3.3 Metabolomic analysis results

**Figure 3:**
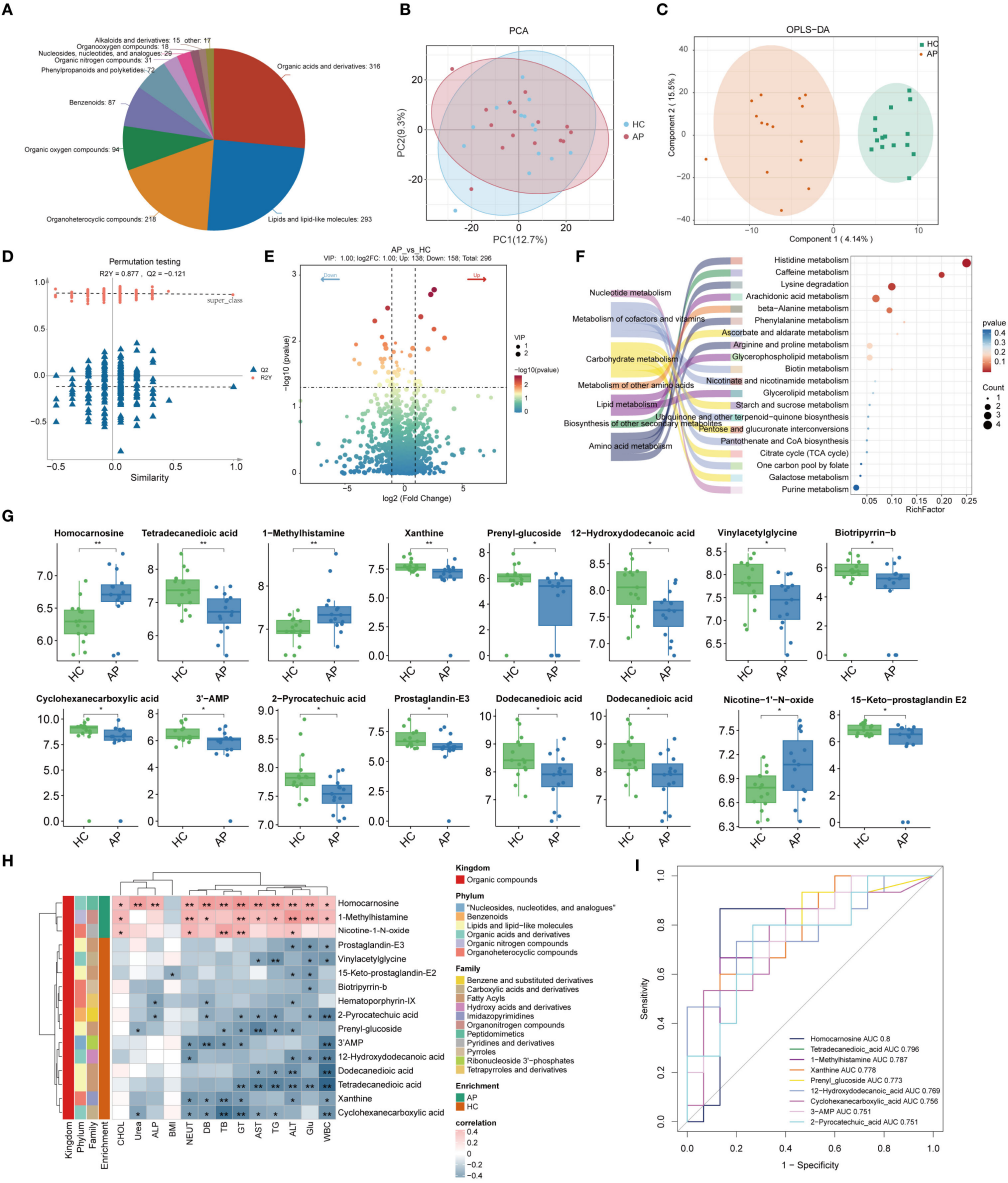
Metabolomic analysis between HC and AP: **(A)** Classification and proportion of identified metabolites across major compound classes. **(B)** PCA scatter plots of metabolomes between HC and AP. **(C, D)** Orthogonal partial least squares discriminant analysis (OPLS-DA) score plot differentiating HC and AP. Permutation test validating the OPLS-DA model with robust R^2^Y and Q^2^, values Q^2^ indicates model predictive ability; values of R^2^Y closer to 1 reflect better model fit. **(E)** Volcano plots of DEMs in HC and AP. The x-axis represents the log2-fold change in metabolites expression between the different groups. The y-axis represents the significance level of the expression difference. **(F)** KEGG Pathway Enrichment Analysis of DEMs. **(G)** The box plots shows the levels of 16 DEMs associated with the occurrence and development of AP in HC and AP. **(H)** Heatmap of correlation between 16 DEMs associated with the occurrence and development of AP and clinical parameters. Red indicates a positive correlation whereas blue indicates a negative correlation (*P < 0.05; **P < 0.01; ***P < 0.001). **(I)** Receiver Operating Characteristic (ROC) Curve analysis of Top Differentially Expressed Metabolites as Potential Biomarkers for AP.

To characterize metabolic alterations in AP, we performed untargeted mass spectrometry (MS)-based profiling of plasma polar metabolites in a subset of patients with AP and HCs. Following rigorous quality control, 1, 604 metabolites were consistently detected across both groups. The major chemical classes included organic acids and derivatives (n = 316), lipids and lipid-like molecules (n = 293), organoheterocyclic compounds (n = 218), organic oxygen compounds (n = 94), and benzenoids (n = 87), among others (**Figure 3A**, **Supplementary Tables 4, S5**). To evaluate the overall metabolic profiles between groups, principal component analysis (PCA) and orthogonal partial least squares discriminant analysis (OPLS-DA) were performed. PCA showed partial overlap between the AP and HC groups, indicating that the global metabolic profiles were not distinctly separated (**Figure 3B**). OPLS-DA demonstrated a visual separation trend between the two groups (R^2^X=0.877, Q^2^Y=-0.121); however, the negative Q² value (Q² = -0.121) suggested limited predictive performance and potential model overfitting. Therefore, the OPLS-DA results were interpreted as exploratory and used primarily for visualization purposes. Differential metabolite identification was subsequently based on combined criteria including VIP scores, fold change, and univariate statistical analysis. At the same time, the samples within each group clustered together tightly reflecting good repeatability of intra-group samples (**Figures 3C, D**). The experimental methods and results of this study demonstrated a high degree of accuracy and reliability.

Differentially expressed metabolites (DEMs) were identified by integrating variable importance in projection (VIP) scores from the OPLS-DA model with univariate statistical criteria (fold change and P-value). A total of 296 DEMs were identified in the AP group compared with HCs, including 138 upregulated and 158 downregulated metabolites (**Figure 3E**, **Supplementary Table 6**). These findings indicate substantial metabolic remodeling in AP. To further investigate the functional relevance of these metabolic alterations, KEGG pathway analysis was performed on the DEMs. Several significantly dysregulated pathways were identified in AP, including amino acid metabolism (ko00340), biosynthesis of other secondary metabolites (ko00232), lipid metabolism (ko00590), and metabolism of other amino acids (ko00410) (**Figure 3F**, **Supplementary Table 7**). These results suggest widespread metabolic dysregulation in AP compared with HCs. Based on literature review and biological relevance, 16 plasma DEMs associated with AP progression were selected for further analysis (**Figure 3G**, **Supplementary Table 8**). Spearman correlation analysis between these metabolites and clinical parameters revealed distinct association patterns (**Figure 3H**). AP-enriched metabolites, including 1-methylhistamine and homocarnosine, showed significant positive correlations with liver enzymes (ALT and AST), glucose (Glu), triglycerides (TG), and white blood cell count (WBC) (P < 0.05), indicating associations with inflammation and metabolic dysfunction. In contrast, HC-enriched metabolites such as tetradecanedioic acid and cyclohexanecarboxylic acid showed consistent negative correlations with these clinical indicators, particularly ALT, AST, and TG, suggesting potential homeostatic roles. Receiver operating characteristic (ROC) analysis was subsequently performed to evaluate the discriminatory potential of these metabolites (**Figure 3I**). Among the 16 metabolites, nine were selected, including homocarnosine, tetradecanedioic acid, 1-methylhistamine, xanthine, prenyl glucoside, 12-hydroxydodecanoic acid, cyclohexanecarboxylic acid, 3’-AMP, and 2-pyrocatechuic acid showed AUC values >0.75. These metabolites may represent candidate exploratory biomarkers for AP and warrant further validation in larger independent cohorts.

### 3.4 Whole-metagenome shotgun sequencing analysis results

**Figure 4:**
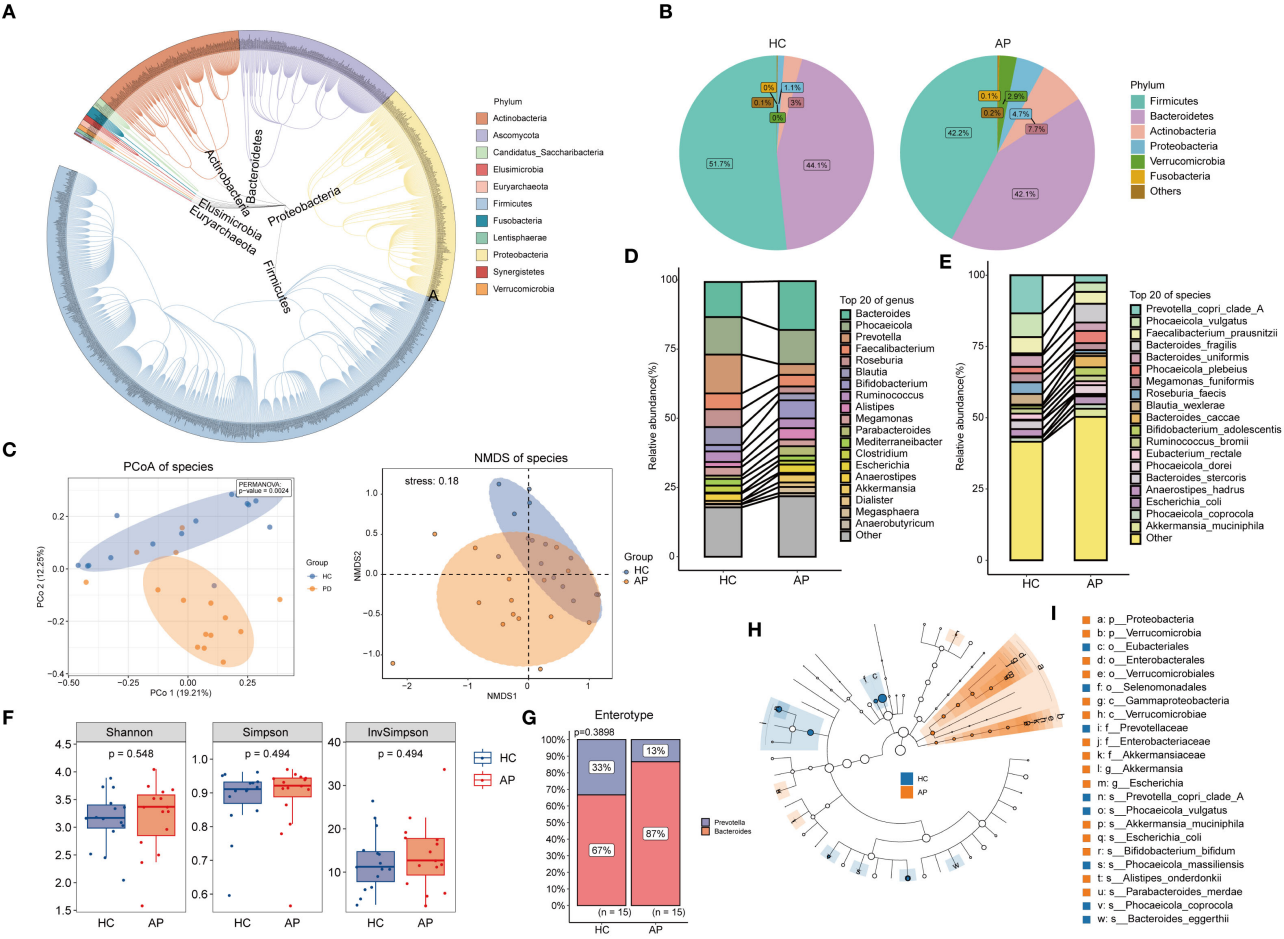
Taxonomic profiling and diversity analysis of gut microbiota in AP: **(A)** Circular cladogram showing the phylogenetic relationships among microbial taxa at the phylum level, colored according to taxonomic classification. **(B, C)** Beta diversity analysis using PCoA and NMDS plots, based on Bray-Curtis dissimilarity, reveals distinct microbial communities between AP and HC. **(D)** Comparison of α-diversity indexes (Shannon, Simpson, and Inverse Simpson) of gut microbiota within HC and AP groups. **(E)** Enterotype analysis reveals altered enterotype distribution in AP patients. **(F)** Pie charts presenting the relative abundance of microbial taxa at the phylum level in HC and AP groups. **(G, H)** Stacked bar diagram represent the relative abundance of top 20 dominant genus and species in the HC and AP groups. **(I)** The cladogram was obtained from the LEfSe analysis (LDA score > 2.0, P < 0.05). The colored circles from inside to out represent the classification level (phylum, class, order, family, and genus). The diameter of each small circle represents their abundance. Yellow nodes represent species with no significant difference and differential gut microflora are colored according to the group. The HC group was shown in blue, and the AP group in red.

To characterize gut microbial alterations in AP, we performed fecal whole-metagenome shotgun sequencing. A total of 615.45 Gb of raw data were generated, yielding 603.81 Gb of clean data after filtering (mean: 20.13 Gb per sample) (**Supplementary Table 9**). Taxonomic profiling identified 12 phyla, 26 classes, 47 orders, 94 families, 336 genera, and 2, 316 species (**Figure 4A**, **Supplementary Table 10**). Alpha diversity (Shannon, Simpson, and InvSimpson indices) showed no significant differences between AP and HC groups (all P > 0.05, **Figure 4D**). In contrast, beta diversity analyses (PCoA and NMDS) revealed significant differences in microbial community structure (PERMANOVA P = 0.0024; **Figures 4B, C**).

At the phylum level, the gut microbiota in both groups was predominantly composed of Bacteroidetes, Firmicutes, Actinobacteria, Proteobacteria, Verrucomicrobia, and Fusobacteria, accounting for the vast majority of total relative abundance (**Figure 4F**). Enterotype analysis showed that both the HC and AP groups were predominantly characterized by a Bacteroides-dominated enterotype. Although the proportion of the Bacteroides enterotype was higher in the AP group than in the HC group (87% vs. 67%), while the Prevotella enterotype showed a corresponding decrease (13% vs. 33%), these differences did not reach statistical significance (P = 0.3898) (**Figure 4E**), suggesting a trend toward altered enterotype distribution in AP. At the genus level, the AP group exhibited significantly reduced abundances of *Prevotella, Blautia, Megamonas, Mediterraneibacter, and Anaerostipes*, alongside increased abundances of *Bacteroides, Bifidobacterium, Alistipes, Parabacteroides, and Escherichia* (**Figure 4G**, **Supplementary Table 11**). Consistent patterns were observed at the species level, where *Prevotella copri, Phocaeicola vulgatus, Faecalibacterium prausnitzii, Bacteroides uniformis*, and *Roseburia faecis* were depleted, whereas *Bacteroides fragilis, Phocaeicola plebeius, Bacteroides caccae, Bifidobacterium adolescentis*, and *Akkermansia muciniphila* were enriched in AP (**Figure 4H**, **Supplementary Table 12**).

**Figure 5:**
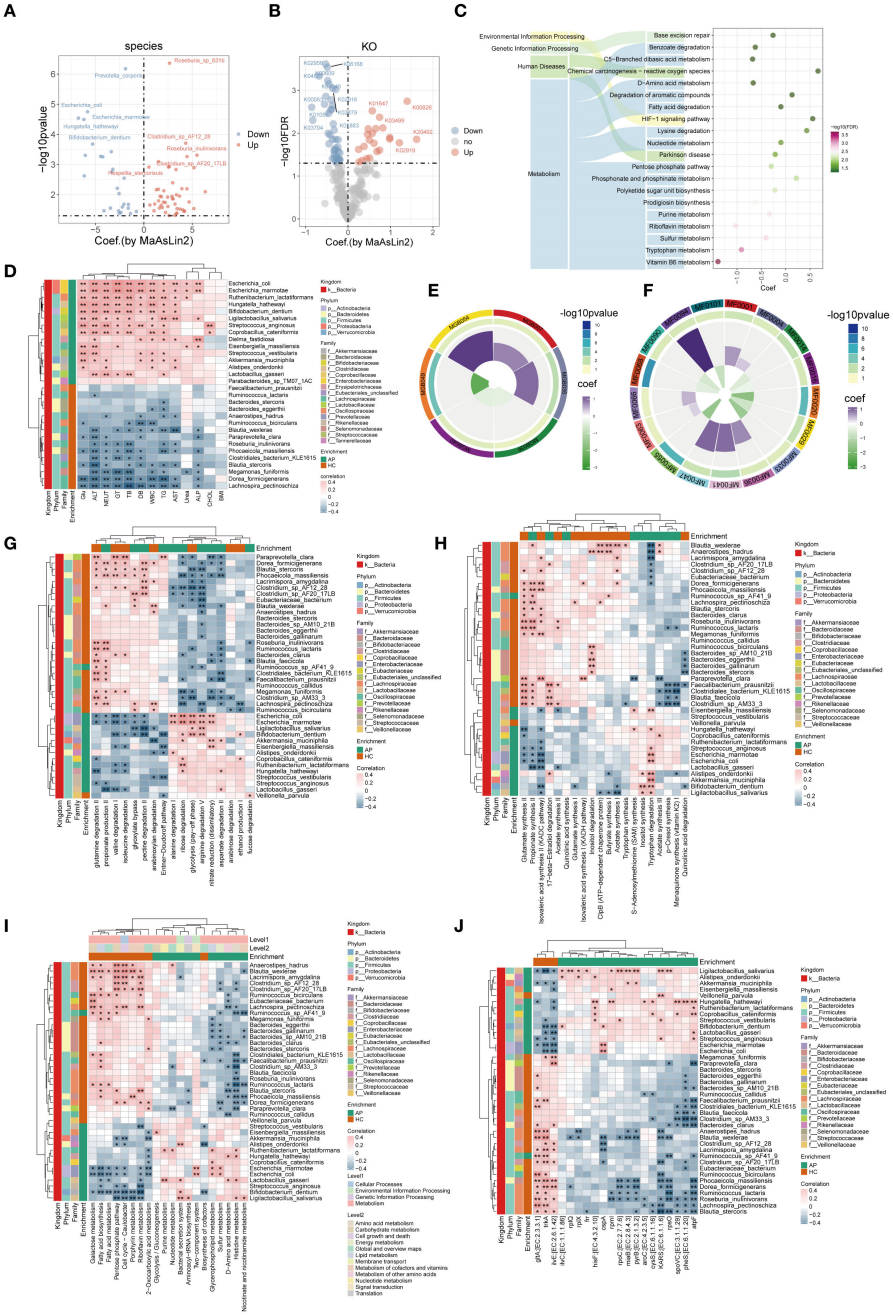
Multi-level functional profiling and host interaction analysis of gut microbiota in AP based on metagenomic sequencing: **(A)** Volcano plot showing differentially abundant microbial species between AP and HC. Red and blue dots represent significantly enriched species in AP and HC, respectively (Q < 0.05). **(B)** Heatmap of Spearman correlations between the differential microbial species and clinical parameters. Red indicates a positive correlation whereas blue indicates a negative correlation(*, P < 0.05; **, P < 0.01; ***, P < 0.001). **(C)** Volcano plot of differentially enriched KEGG orthologs (KOs) between AP and HC. Blue points indicate down-regulated genes, red points indicate up-regulated genes, and gray points indicate genes with no significant differential expression. **(D)** KEGG pathway-level functional enrichment of altered microbial genes in AP. Bubble size indicates the number of genes mapped per pathway, and color denotes enrichment significance. **(E, F)** Differentially enriched gut-brain modules (GBMs) and gut-metabolite modules (GMMs) between AP and HC. Bar plots show modules significantly enriched or depleted in AP compared to HC. Each bar represents a specific module, with bar length indicating the effect size (regression coefficient) and color denoting the statistical significance. **(G–J)** Heatmaps displaying Spearman correlations between representative microbial species and **(G)** gut-metabolite modules (GMMs), **(H)** gut-brain modules (GBMs), **(I)** KEGG pathways, and **(J)** KEGG orthologs (KOs). Color scale represents correlation coefficients, with red indicating positive and blue indicating negative correlations. Asterisks denote statistical significance (*, P < 0.05; **, P < 0.01; ***, P < 0.001).

LEfSe analysis further identified key discriminatory taxa. Species enriched in the AP group included *Streptococcus anginosus, Escherichia coli*, and *Ligilactobacillus salivarius*, whereas taxa such as *Megamonas funiformis, Lachnospira pectinoschiza, Blautia wexlerae*, and *Dorea formicigenerans* were more abundant in HCs (**Figure 4I**; **Supplementary Figure 1**; **Supplementary Table 13**). These findings collectively indicate a shift toward enrichment of pro-inflammatory or opportunistic taxa and depletion of beneficial commensals in AP. Overall, 74 differentially abundant species were identified, including 48 enriched and 26 depleted in AP (**Figure 5A**; **Supplementary Tables 14**, **15**). A random forest model further identified key microbial signatures, with *Streptococcus anginosus, Megamonas funiformis*, and *Lachnospira pectinoschiza* among the top contributors to classification performance (**Supplementary Figures 2, 3**). These taxa may serve as potential microbial biomarkers for distinguishing AP from healthy controls.

To explore the functional implications of gut microbial alterations in AP, we analyzed differences in Kyoto Encyclopedia of Genes and Genomes (KEGG) orthology (KO) genes, metabolic pathways, gut-brain modules (GBMs), and gut-metabolite modules (GMMs) between AP patients and HCs. A total of 64 differential KO genes were identified, including 18 enriched and 42 depleted in AP (**Figure 5C**, **Supplementary Table 16**). The top five differential KO genes included K02916 (RP-L35), K02956 (RP-S15), K02879 (RP-L17), K03046 (rpoC), and K06168 (miaB). KEGG functional analysis showed that AP-associated microbiota were enriched in pathways related to amino acid metabolism (including D-amino acid and lysine degradation), nucleotide metabolism, fatty acid degradation, and HIF-1 signaling. In contrast, pathways involved in vitamin B6 metabolism and tryptophan metabolism were significantly depleted (**Figure 5D**), suggesting disruptions in microbial metabolic function and host-microbiota interactions. Correlation analysis between differential microbial species and clinical phenotypes revealed distinct associations (**Figure 5B**). AP-enriched species, including *Escherichia coli, Ruthenibacterium lactatiformans*, and *Streptococcus anginosus*, were positively correlated with glucose (Glu), neutrophil percentage (%NEUT), and total bilirubin (TB) (P < 0.05). In contrast, HC-enriched species such as *Ruminococcus bicirculans, Paraprevotella clara*, and *Roseburia inulinivorans* were negatively correlated with ALT, AST, and serum urea, suggesting potential associations with metabolic homeostasis. Further functional analysis identified three significantly enriched and three significantly depleted gut-brain modules in AP (**Figure 5E**). Enriched GBMs included MGB035 (isovaleric acid synthesis II), MGB007 (glutamate synthesis II), and MGB054 (propionate synthesis II), whereas MGB049 (tryptophan degradation), MGB040 (menaquinone synthesis I), and MGB036 (S-adenosylmethionine synthesis) were depleted. In addition, 17 gut-metabolite modules were differentially enriched between groups (**Figure 5F**). Modules involved in ribose degradation (MF0020) and arginine degradation V (MF0055) were depleted in AP, whereas propionate production II (MF0094) and glutamine degradation II (MF0047) were enriched. These findings indicate substantial functional remodeling of microbial metabolic capacity in AP. We further evaluated associations between differential microbial taxa and microbial functional features. At the KO level, HC-enriched taxa, including *Faecalibacterium prausnitzii* and *Lachnospira pectinoschiza*, were positively associated with genes involved in energy production and amino acid metabolism (e.g., gltA, ilvE, and trkA). In contrast, AP-enriched species such as *Escherichia coli* and *Ligilactobacillus salivarius* were positively correlated with stress-response and translational genes (e.g., cspA and rpsO), suggesting adaptive responses to inflammatory stress (**Figure 5J**). At the pathway level, HC-associated microbes were primarily linked to fatty acid biosynthesis and pentose phosphate metabolism, whereas AP-enriched taxa were more strongly associated with glycerophospholipid metabolism and sulfur metabolism, indicating distinct metabolic functional preferences (**Figure 5I**). GBM analysis showed that AP-associated microbes were correlated with pathways related to tryptophan degradation and S-adenosylmethionine synthesis, whereas HC-enriched taxa were linked to aromatic amino acid degradation and glycolysis pathways (**Figure 5H**). Similarly, GMM analysis demonstrated that AP-associated microbes were positively correlated with alanine, ribose, and arginine degradation modules, while HC-associated species were associated with glycolysis and aromatic amino acid metabolism pathways that may support metabolic homeostasis (**Figure 5G**). Collectively, these findings demonstrate that gut microbial alterations in AP extend beyond compositional shifts and involve substantial functional remodeling that may contribute to inflammatory activation and metabolic dysregulation.

### 3.5 Construction of multi-omics correlation network

**Figure 6:**
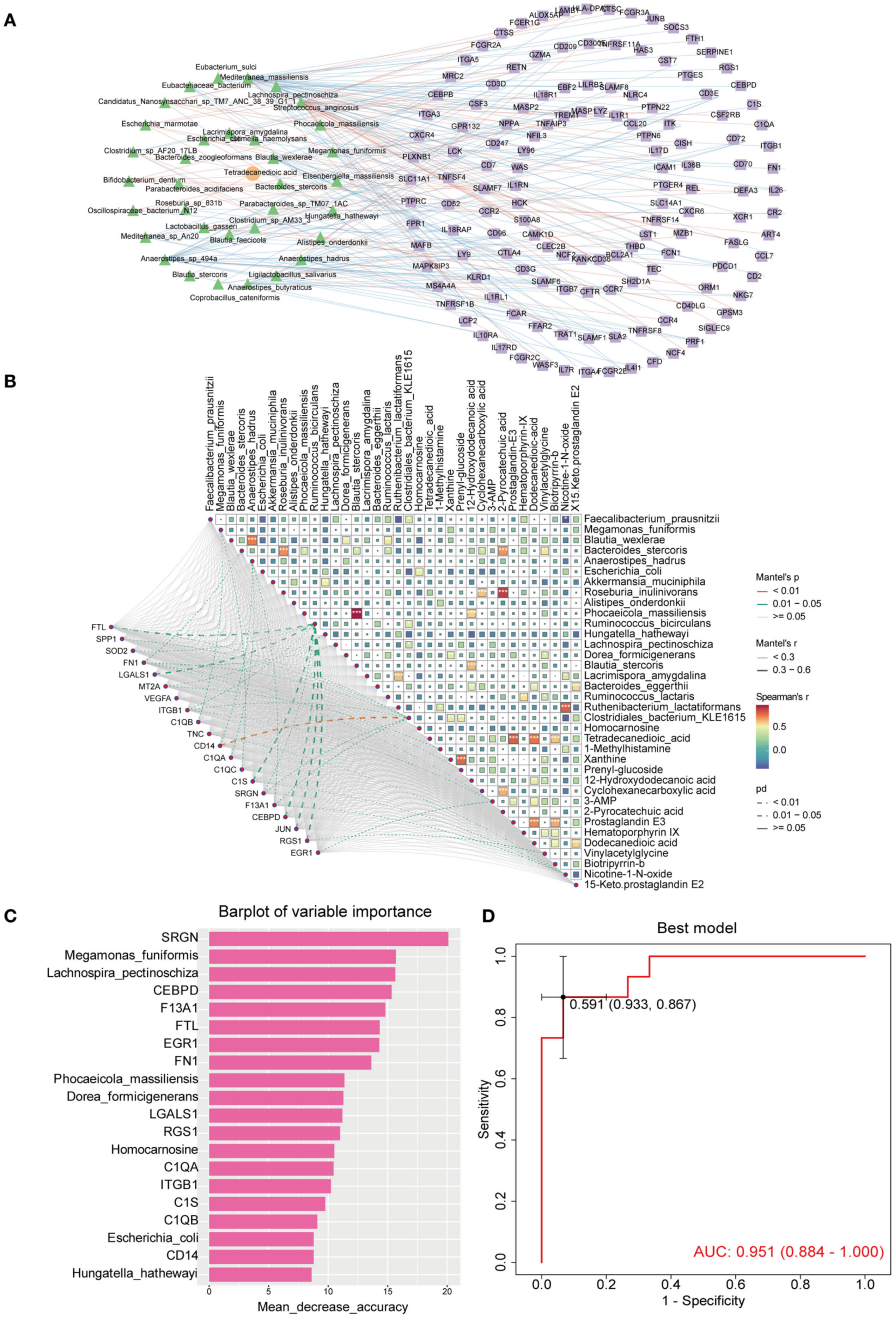
Integrative multi-omics network based on Spearman correlation and constructing a multivariable linear regression model. **(A)** Correlation Network among immune-related DEGs, DEMs, and differential gut microbiota. Only correlations associated with the absolute value of Spearman correlation coefficient>0.6 are presented in the network. The thicker the line, the stronger the correlation. **(B)** Correlation heatmap, among immune related DEGs, DEMs, and differential gut microbiota, based on the mantel test. The upper right triangle represents the relationship between differential metabolites and differential gut microbiota (a total of 36 variables). The color gradient indicates the Spearman correlation coefficient. Red and blue denote positive and negative correlations, respectively. Darker colors or larger rectangle areas indicate higher absolute correlation coefficients. Asterisks denote the significance of the correlation. The connecting lines in the middle represent the relationship between immune-related differential genes and the other 36 variables. Line color indicates the range of P-value, solid lines represent positive correlation coefficients, and line width indicates the magnitude of Mantel’s (r) **(C)** The plot displays the importance ranking of 56 variables in the random forest model of AP. The abscissa represents the importance value, and the ordinate represents the variable name. **(D)** AUC distribution plot of the random forest model. The random forest model incorporates the top-ranking 3 variables with the best AUC of 0.951.

To investigate cross-omics interactions, Spearman correlation analysis was performed among immune-related DEGs, DEMs, and DGMs. Significant associations were defined as correlation coefficients >0.6 or <−0.6 with P < 0.05 and were visualized as an integrative multi-omics network (**Figure 6A**, **Supplementary Table 17**). A total of 215 significant correlations were identified, with microbe-gene interactions accounting for the largest proportion, followed by gene-metabolite and microbe-metabolite associations, suggesting a prominent role of gut microbiota in host immune regulation. AP-enriched species such as *Escherichia coli* and *Streptococcus anginosus* were positively correlated with pro-inflammatory genes, including CD36, CTLA4, and CCR7. In contrast, HC-enriched taxa such as *Faecalibacterium prausnitzii* and *Blautia wexlerae* were associated with immune-regulatory genes, including IL10RA and SOCS3. Key metabolites, including tetradecanedioic acid and cyclohexanecarboxylic acid, appeared to function as intermediates linking microbial alterations with host immune gene expression. To further investigate potential regulatory interactions, we performed a Mantel test-based tripartite correlation analysis integrating the top 20 immune-related DEGs, top 20 differential microbial species, and 16 significantly altered metabolites (**Figure 6B**). Overall, AP-enriched microbial species tended to cluster with pro-inflammatory metabolites and immune-related genes, whereas HC-enriched commensals were more frequently associated with metabolites linked to metabolic homeostasis. For example, the HC-enriched species *Ruminococcus bicirculans* showed positive correlations with host genes including SOD2, VEGFA, and FN1. In contrast, the AP-enriched species *Escherichia coli*, and *Ruthenibacterium lactatiformans* exhibited negative correlations with immune-related genes such as CD14, LGALS1, and F13A1. These microbial-gene interactions were further linked to metabolites including xanthine, and 2-pyrocatechuic acid. Collectively, These findings highlight a tightly interlinked network whereby specific gut microbes and their associated metabolites may cooperatively regulate immune-related gene expression, potentially shaping the host inflammatory milieu under disease conditions. To further prioritize candidate exploratory biomarkers, a random forest model was constructed using 56 candidate features derived from the multi-omics interaction network. The model achieved its highest AUC of 0.951 when three features were included (**Figure 6C**). The optimal feature combination consisted of *Lachnospira pectinoschiza*, *Megamonas funiformis*, and SRGN. These features may represent candidate exploratory biomarkers for AP, although further validation in independent cohorts is required.

### 3.6 Experimental validation of key candidate biomarkers

**Figure 7:**
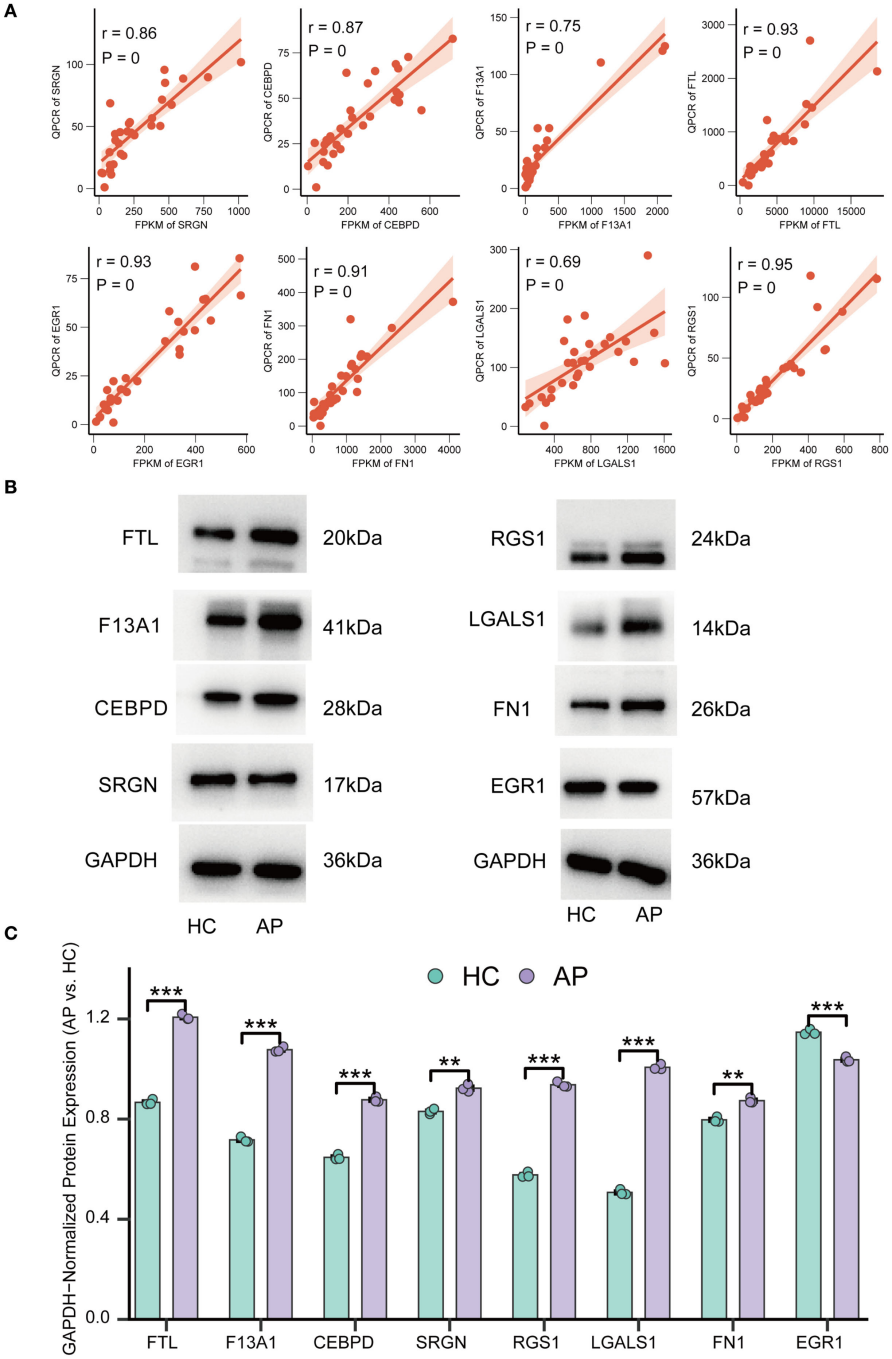
Experimental validation of key genes identified by RNA sequencing. **(A)** qRT-PCR verification eight selected genes (FTL, F13A1, CEBPD, SRGN, RGS1, LGALS1, FN1, and EGR1) showing consistent expression patterns with transcriptomic data. **(B)** Western blot analysis of corresponding proteins in AP and HC samples. **(C)** Quantitative analysis of protein expression levels normalized to GAPDH, with statistical significance indicated (*P < 0.05, **P < 0.01, ***P < 0.001).

To assess the reliability of the transcriptomic findings, qRT-PCR was performed for eight candidate genes (FTL, F13A1, CEBPD, SRGN, RGS1, LGALS1, EGR1, and FN1). As shown in **Figure 7A**, the mRNA expression levels measured by qRT-PCR were highly consistent with the RNA-seq data (FPKM values), with correlation coefficients (r) ranging from 0.69 to 0.97 (all P < 0.05), supporting the robustness of the transcriptomic results.

We further evaluated the protein expression of these candidates in plasma samples using Western blot analysis. Consistent with the transcriptional findings, protein levels of FTL, F13A1, CEBPD, SRGN, RGS1, LGALS1, and FN1 were significantly elevated in the AP group compared with the HC group, whereas EGR1 protein expression was significantly reduced (P < 0.01) (**Figures 7B, C**). Collectively, these findings support the consistency of the identified molecular alterations at both the transcriptomic and protein levels in acute pancreatitis.

## 4 Discussion

Acute pancreatitis (AP) is a complex inflammatory disease characterized by abrupt onset, heterogeneous clinical manifestations, and limited targeted therapeutic options. In the present study, we applied an integrative multi-omics strategy combining transcriptomic, metabolomic, and metagenomic analyses to comprehensively characterize the molecular landscape of AP. Our findings demonstrate that AP is characterized by coordinated immune activation, metabolic reprogramming, and gut microbial dysbiosis, which together form an interconnected regulatory network that may contribute to disease progression. Specifically, we identified extensive inflammatory activation signatures, substantial metabolic disturbances, and functional remodeling of the gut microbiota, highlighting the complex interplay between host immunity, metabolism, and microbial ecology in AP. Notably, integrative multi-omics analysis further revealed coordinated associations among immune-related genes, metabolites, and microbial species, suggesting potential host-microbiota-metabolism interaction axes involved in AP pathogenesis. In addition, we identified several candidate exploratory biomarkers with promising discriminatory performance, although these findings should be interpreted cautiously and require further validation in larger independent cohorts. Collectively, our study provides a more comprehensive perspective on the systemic nature of AP and highlights the potential value of multi-omics integration in improving the understanding of complex inflammatory diseases, particularly where single-omics approaches may offer only limited insights.

### 4.1 Immune dysregulation and inflammatory activation in acute pancreatitis

Transcriptomic profiling revealed extensive activation of inflammatory pathways in AP, particularly those involved in immune and inflammatory signaling, including NF-κB, IL-17, and cytokine-cytokine receptor interaction pathways (15, 17). These findings are consistent with previous studies highlighting the pivotal role of dysregulated immune activation in AP pathogenesis. Excessive activation of these pathways may promote neutrophil recruitment, pro-inflammatory cytokine release, and amplification of local pancreatic injury. In addition to pro-inflammatory signaling, we observed downregulation of regulatory pathways such as MAPK and apoptosis, suggesting an imbalance between inflammatory activation and resolution. This dysregulation may contribute to sustained inflammatory cascades, impaired clearance of activated immune cells, and progression toward more severe disease states (16).

We further identified 409 immune-related DEGs, and PPI network analysis revealed several central hub genes involved in cytokine signaling and immune cell recruitment. These findings are consistent with previous reports demonstrating that excessive neutrophil infiltration and cytokine storms contribute to pancreatic tissue injury and organ dysfunction in AP (17, 28). Collectively, these results support the notion that immune dysregulation is a central feature of AP pathophysiology and may represent an important therapeutic target (11).

### 4.2 Systemic metabolic reprogramming reflects organ dysfunction and inflammatory stress

Plasma metabolomic profiling identified 296 significantly altered metabolites in AP, indicating substantial disruptions in amino acid metabolism, lipid metabolism, and energy homeostasis. Several AP-enriched metabolites, including xanthine and 1-methylhistamine, showed positive correlations with clinical indicators of liver injury and systemic inflammation, such as elevated transaminases and leukocyte counts. These metabolites may reflect enhanced purine metabolism and histamine-related inflammatory responses, which have been implicated in oxidative stress and inflammatory signaling (8). In contrast, metabolites enriched in HCs, such as tetradecanedioic acid and cyclohexanecarboxylic acid, showed negative correlations with inflammatory markers and liver enzymes. These findings suggest that these metabolites may be associated with metabolic homeostasis in healthy individuals (29, 30). Pathway enrichment analysis further demonstrated that key immunometabolic axes were dysregulated in AP, notably those related to glutamine and arginine metabolism. These pathways are central to immune cell proliferation, nitric oxide production, and redox homeostasis, and their disturbance may contribute to immune cell exhaustion and impaired host defense (31, 32). Additionally, perturbations in fatty acid β-oxidation and branched-chain amino acid metabolism highlight the metabolic inflexibility and catabolic stress characteristic of AP (33).

Our findings are consistent with previous metabolomic studies reporting metabolic disturbances and oxidative stress in AP. Importantly, by integrating metabolomic data with transcriptomic profiles, we further identified significant associations between differential metabolites and immune-related genes, highlighting potential links between metabolic remodeling and immune activation. Moreover, receiver operating characteristic (ROC) curve analysis identified several differential metabolites with promising discriminatory performance (AUC > 0.75). By integrating metabolomic alterations with immune-related transcriptional signatures, our study provides a broader framework for understanding the interplay between metabolic dysregulation and immune activation in AP. Although these metabolites may represent candidate exploratory biomarkers, further validation in larger independent cohorts is required before potential clinical application. Collectively, these findings highlight the potential contribution of immunometabolic dysfunction to AP pathogenesis and may inform future biomarker research.

### 4.3 Gut microbiota dysbiosis and functional remodeling in acute pancreatitis

Metagenomic sequencing revealed substantial alterations in the gut microbiome of patients with AP compared with HCs. Specifically, AP patients exhibited increased abundance of potential pathobionts, including *Escherichia coli* and *Streptococcus anginosus*, alongside depletion of beneficial commensals such as *Faecalibacterium prausnitzii* and *Ruminococcus bicirculans*, which is consistent with previous studies (11, 34, 35). These compositional shifts suggest that gut microbial dysbiosis may contribute to the inflammatory and metabolic disturbances observed in AP. Functional profiling further demonstrated marked microbial remodeling. KEGG ortholog analysis revealed enrichment of microbial genes involved in amino acid degradation, nucleotide metabolism, fatty acid degradation, and HIF-1 signaling pathways, which may reflect microbial adaptation to inflammatory and hypoxic conditions (13, 36). In contrast, pathways involved in vitamin B6 metabolism and tryptophan metabolism were reduced, suggesting impaired microbial metabolic functions that may influence host homeostasis (37).

Furthermore, both gut-brain modules (GBMs) and gut-metabolite modules (GMMs) showed significant alterations. AP-enriched GBMs included glutamate synthesis and propionate production pathways, while those for tryptophan degradation, S-adenosylmethionine (SAM) synthesis, and menaquinone (vitamin K2) were reduced. These functional shifts suggest that AP-associated microbes may promote neuroinflammation and metabolic stress via disrupted microbial-neuroendocrine crosstalk (38). Similarly, GMMs associated with glutamine and alanine degradation were enriched in AP, whereas glycolysis and aromatic amino acid degradation-typically supporting mucosal and metabolic integrity-were predominant in HCs (39, 40).

Correlational analysis further supported these findings. AP-enriched species such as *Escherichia coli, Ruthenibacterium lactatiformans*, and *Streptococcus anginosus* positively correlated with systemic inflammation markers (e.g., Glu, NEUT, TB), while HCs-associated taxa like *Ruminococcus bicirculans, Paraprevotella clara*, and *Roseburia inulinivorans* were negatively associated with ALT, AST, and serum urea, suggesting potential links to metabolic homeostasis. At the gene level, AP-associated microbes were linked to translational stress and inflammation-related genes (e.g., rpsO, cspA), whereas HCs taxa were associated with energy and amino acid metabolism genes (e.g., gltA, ilvE). Importantly, multiple microbial pathways involved in short-chain fatty acid (SCFA) biosynthesis were also altered. While SCFAs such as butyrate are typically considered anti-inflammatory and beneficial for gut health (41–43), their role in AP appears context-dependent. Our findings are in line with Ammer-Herrmenau et al. (14), who observed increased SCFA-producing taxa in severe AP, and highlight the paradox observed in the PROPATRIA trial (44, 45), where probiotic supplementation led to worsened outcomes. These inconsistencies suggest that SCFA effects may depend on strain-specific activity, gut niche (luminal vs mucosal), and the host inflammatory milieu.

Taken together, these data demonstrate that gut microbiota in AP are not only compositionally disrupted but also functionally reprogrammed, displaying enhanced pro-inflammatory capacity, impaired support for host metabolism and immunity, and potential promotion of neuroinflammatory signaling. In addition, our multi-omics correlation analyses revealed close associations among microbial taxa, host immune gene expression, and circulating metabolites. For example, *Escherichia coli* was positively associated with pro-inflammatory genes such as CCR7 and CD36, whereas commensal taxa such as *Faecalibacterium prausnitzii* showed inverse associations with systemic inflammatory markers. These findings further support the concept that gut microbiota may influence AP progression through complex host-microbe-metabolite interactions (46, 47).

### 4.4 Multi-omics integration reveals host-microbiome interactions in acute pancreatitis

By constructing an integrative correlation network across differentially expressed genes (DEGs), differentially expressed metabolites (DEMs), and differentially abundant microbial taxa (DGMs), we identified multiple coordinated interactions linking gut microbiota, host immunity, and metabolism. Notably, immune-related genes such as CD36, CTLA4, and CCR7-known mediators of lipid uptake, T-cell regulation, and chemokine-directed migration-were strongly associated with AP-enriched microbial species, including *Escherichia coli* and *Streptococcus anginosus (*48–50). These microbial taxa were also correlated with inflammatory metabolites such as xanthine and 1-methylhistamine, suggesting potential involvement in enhanced innate immune activation and oxidative stress responses. In contrast, HC-enriched taxa such as *Faecalibacterium prausnitzii* and *Blautia wexlerae* were associated with immune-regulatory genes including IL10RA and SOCS3, as well as metabolites such as tetradecanedioic acid, which may contribute to anti-inflammatory signaling and maintenance of mucosal homeostasis. Collectively, these findings suggest the presence of coordinated immunometabolic disturbances in AP, in which disruption of host-microbiota interaction networks may contribute to systemic inflammation.

Further integrative correlation analysis identified several interconnected microbial, metabolic, and immune-related features. For example, SRGN, tetradecanedioic acid, and *Ruminococcus bicirculans* exhibited significant associations within the multi-omics network, suggesting a potential interaction pattern linking host immune responses, metabolic alterations, and gut microbial changes. These findings are generally consistent with previous studies demonstrating that gut microbiota-derived metabolites may influence immune responses and disease severity in AP (24, 51), supporting the concept that AP pathogenesis likely involves complex host-microbiota-metabolism interactions rather than isolated molecular alterations.

Compared with Liu et al. (8), who reported microbiota-associated plasma lipid alterations in hyperlipidemic AP, our findings further expand the link between gut microbial dysbiosis and systemic metabolic disturbances across a broader spectrum of AP phenotypes. In addition, our exploratory random forest analysis identified SRGN, *Lachnospira pectinoschiza, and Megamonas funiformis* as a potential multi-omics feature set with promising discriminatory performance (AUC = 0.951). However, given the limited sample size and absence of external validation, these findings should be interpreted cautiously and require confirmation in larger independent cohorts.

### 4.5 Clinical relevance and future perspectives

Our findings may provide additional context for several unresolved questions in AP management. For example, the failed PROPATRIA probiotic trial-previously attributed largely to probiotic-related complications-may also reflect the complex and context-dependent roles of gut microbial functions, as our data suggest that both beneficial and potentially harmful SCFA-related pathways may coexist depending on microbial composition and host inflammatory status. In addition, the identification of microbial, metabolic, and immune-associated features may help inform future biomarker research aimed at improving early disease assessment. However, these findings remain exploratory and require validation in larger prospective cohorts. Our results also highlight the potential importance of host-microbiome-metabolite interactions in AP pathogenesis, which may provide a foundation for future studies investigating microbiome-targeted interventions, including dietary modulation, prebiotics, probiotics, or metabolite-based therapies. Further mechanistic and clinical studies are needed before these findings can be translated into therapeutic strategies.

## 5 Limitations and future directions

Several limitations of this study should be acknowledged. First, the relatively small sample size and single-center design may limit the generalizability of our findings. In addition, acute pancreatitis is a highly heterogeneous disease, and important clinical factors-including disease etiology, disease severity, metabolic status, treatment exposure, and the interval from symptom onset to sample collection-may influence transcriptomic, metabolomic, and microbiome profiles. Although age, sex, and BMI were adjusted in the microbiome analysis, residual confounding across all three omics layers may still exist. Due to the limited sample size, adequately powered stratified analyses could not be performed. Second, the cross-sectional design of this study precludes causal inference regarding the observed host immune-metabolic-microbial associations. Therefore, the identified relationships should be interpreted as associative rather than mechanistic. Longitudinal studies with multi-timepoint sampling are needed to better characterize the dynamic interactions among these systems during AP progression and recovery. Third, although qRT-PCR and Western blot analyses provided experimental support for several candidate biomarkers, these validation experiments were conducted in a relatively limited cohort and require confirmation in larger independent populations. Finally, functional validation of the identified candidate biomarkers and microbial taxa remains necessary. Future studies should expand sample size, incorporate longitudinal multi-timepoint sampling, and perform mechanistic investigations in experimental models. Approaches such as fecal microbiota transplantation and targeted microbial colonization may help further clarify the potential roles of candidate microbial taxa in host immune and metabolic regulation during acute pancreatitis.

## 6 Conclusion

Our integrative multi-omics analysis revealed coordinated alterations in immune responses, metabolic pathways, and gut microbial communities in acute pancreatitis. We identified candidate microbial and metabolic biomarkers associated with AP and characterized potential host-microbiome interaction patterns that may contribute to disease heterogeneity. These findings provide exploratory insights into the complex immune-metabolic-microbial landscape of AP and highlight the potential utility of multi-omics approaches for future biomarker discovery and mechanistic investigations in inflammatory diseases.

## Data Availability

The datasets presented in this study can be found in online repositories. The names of the repository/repositories and accession number(s) can be found in the article/**Supplementary Material**.
