# Supplementary figures and images for: Integrative multi-omics profiling reveals coordinated immunometabolic reprogramming and host-microbiome interactions in acute pancreatitis
Source: Front Immunol. 2026 Jun 19;17:1828633. doi: 10.3389/fimmu.2026.1828633 (PMC13328029; doi:10.3389/fimmu.2026.1828633)

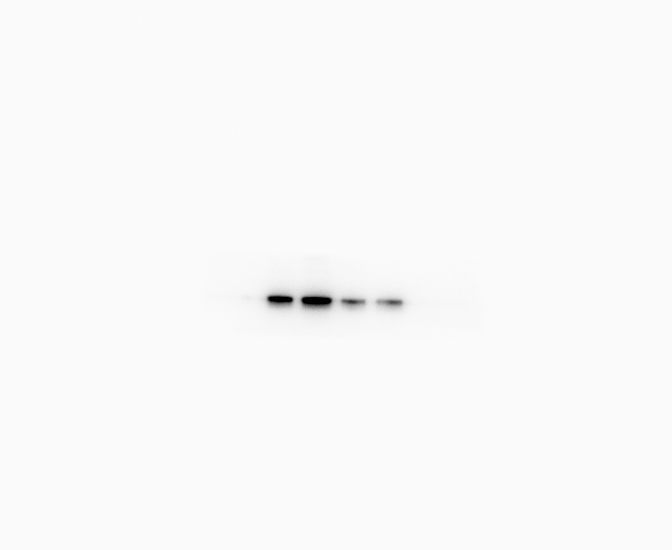

Supplement: Supplementary file 1 [file DataSheet1.zip › Raw Western blot images/CEBPD.tif]

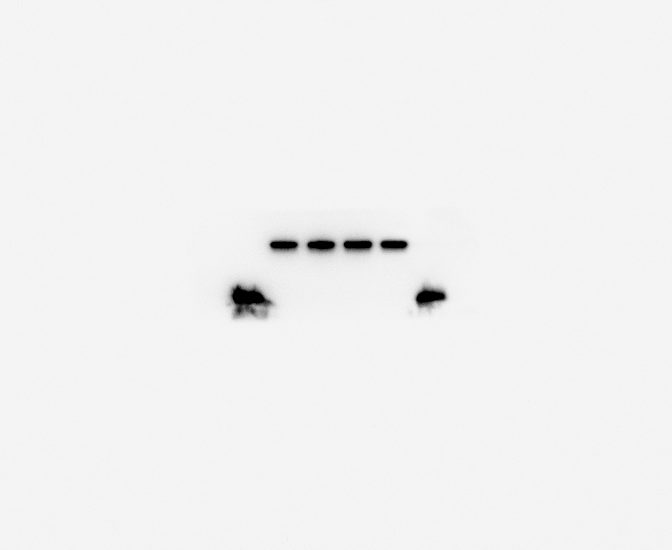

Supplement: Supplementary file 1 [file DataSheet1.zip › Raw Western blot images/EGR1.tif]

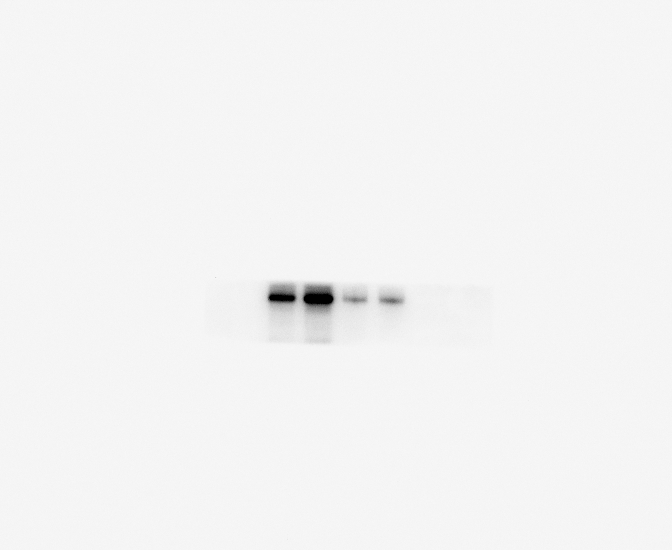

Supplement: Supplementary file 1 [file DataSheet1.zip › Raw Western blot images/F13A1.tif]

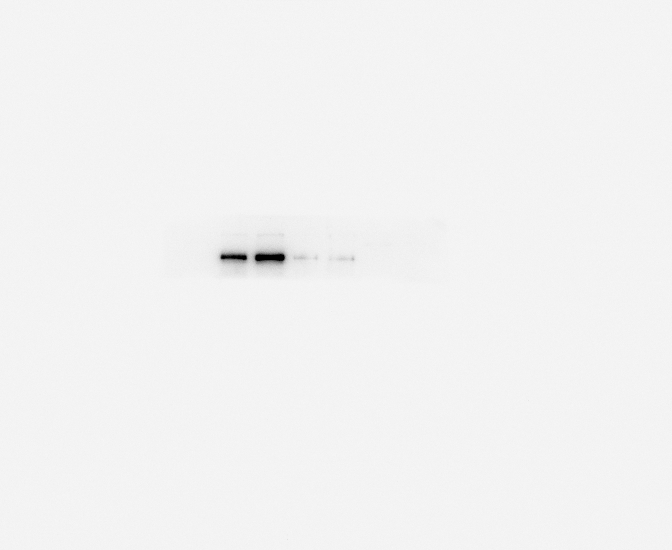

Supplement: Supplementary file 1 [file DataSheet1.zip › Raw Western blot images/FN1.tif]

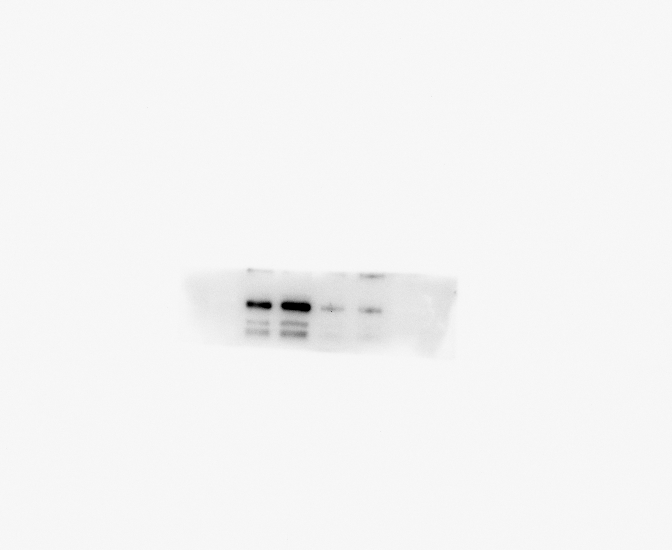

Supplement: Supplementary file 1 [file DataSheet1.zip › Raw Western blot images/FTL.tif]

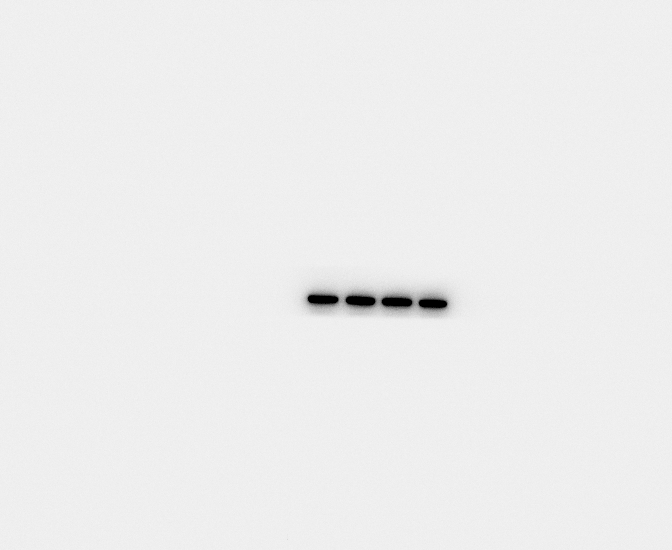

Supplement: Supplementary file 1 [file DataSheet1.zip › Raw Western blot images/GAPDH-1.tif]

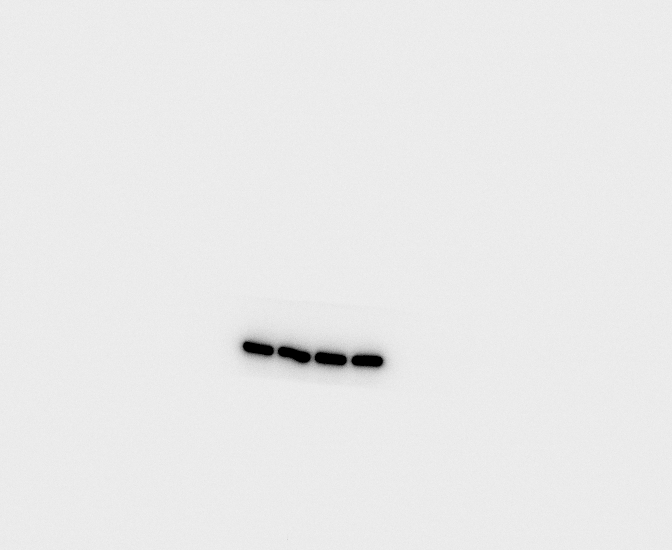

Supplement: Supplementary file 1 [file DataSheet1.zip › Raw Western blot images/GAPDH2.tif]

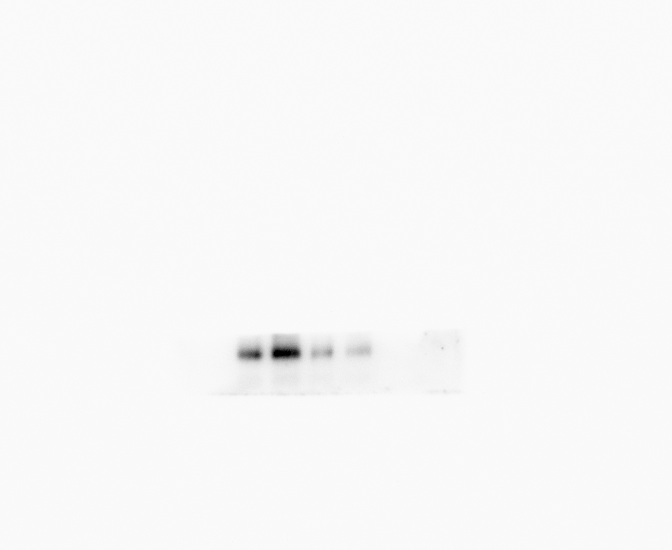

Supplement: Supplementary file 1 [file DataSheet1.zip › Raw Western blot images/LGALS1.tif]

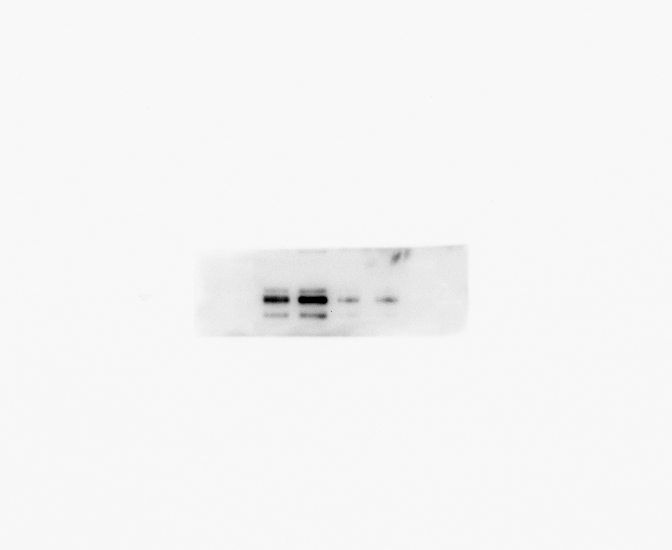

Supplement: Supplementary file 1 [file DataSheet1.zip › Raw Western blot images/RGS1.tif]

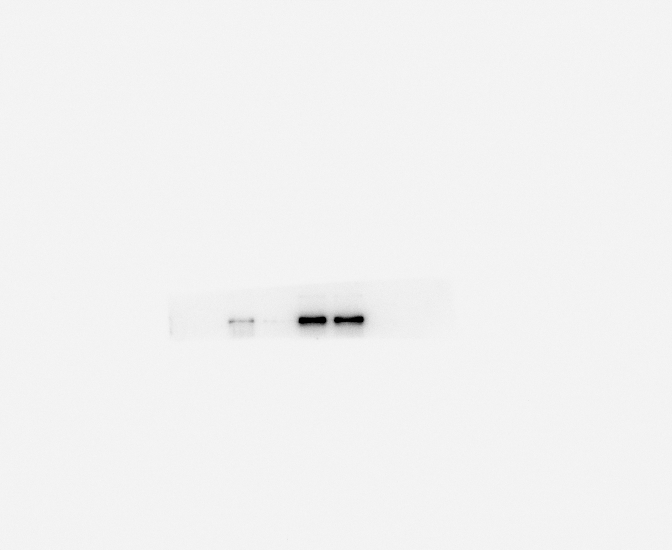

Supplement: Supplementary file 1 [file DataSheet1.zip › Raw Western blot images/SRGN.tif]
