# Supplementary material for: Integrative multi-omics profiling reveals coordinated immunometabolic reprogramming and host-microbiome interactions in acute pancreatitis
Source: Front Immunol. 2026 Jun 19;17:1828633. doi: 10.3389/fimmu.2026.1828633 (PMC13328029; doi:10.3389/fimmu.2026.1828633)
Supplement: Supplementary file 2 [file DataSheet2.docx]

Supplementary Material

# Supplementary Data

## Supplementary Figures


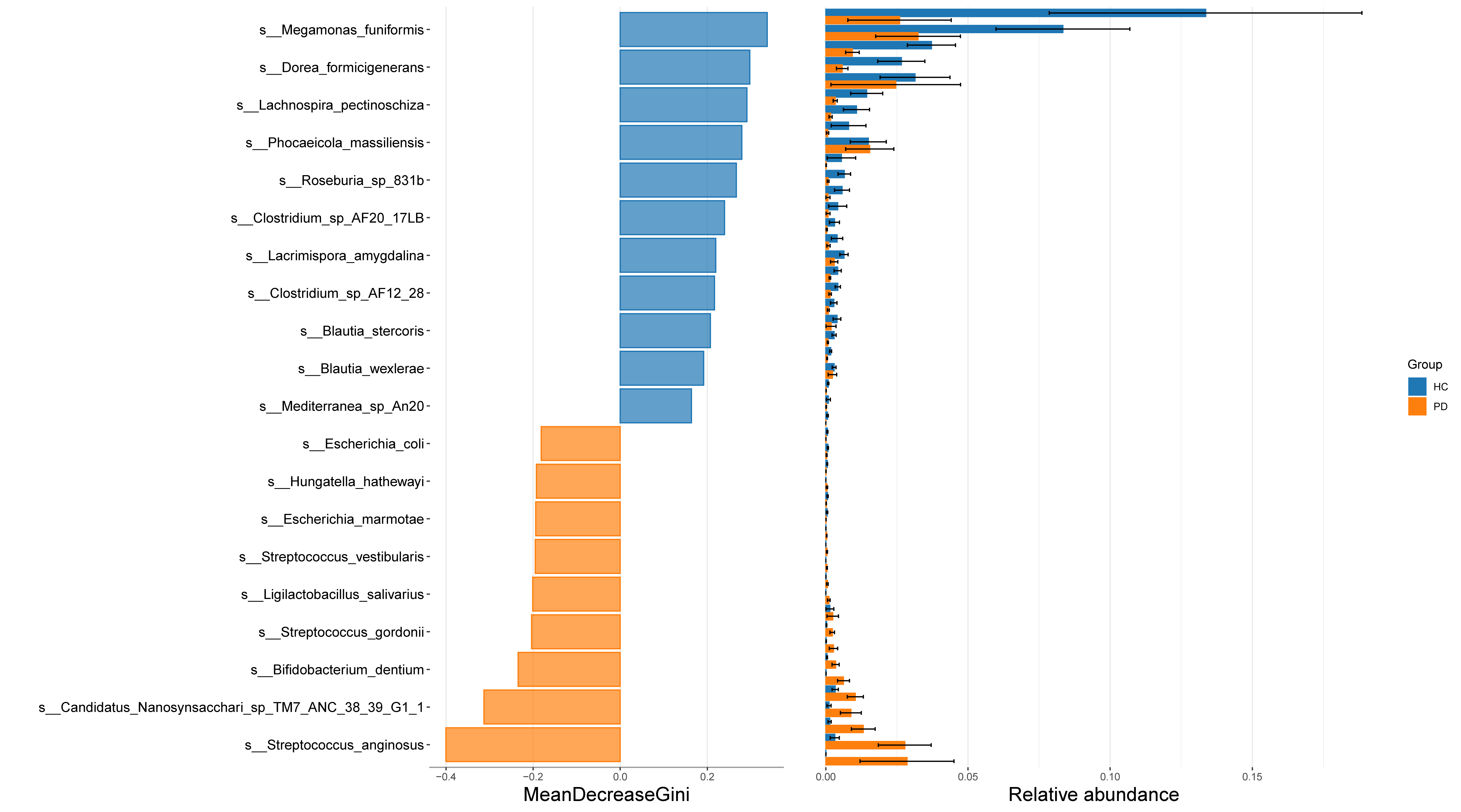


#### **Supplementary Figure 1.** LEfSe analysis identifying differential microbial species between AP and HC groups. The left panel shows the top discriminative species ranked by mean decrease in Gini index from a random forest model, while the right panel displays their relative abundance across groups. Species enriched in the AP group are colored in orange, and those enriched in the HC group are colored in blue.


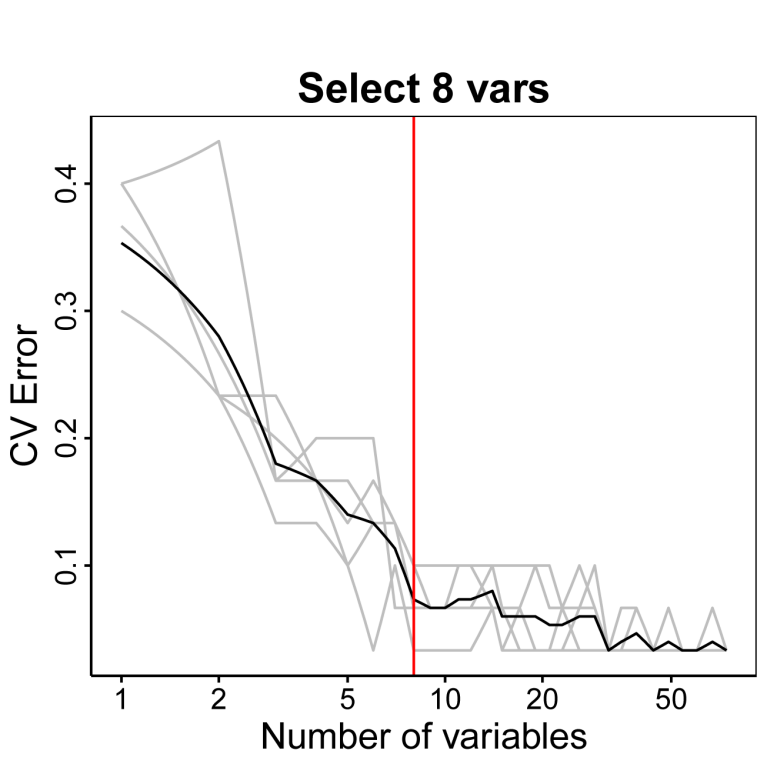


#### **Supplementary Figure 2.** Cross-validation error curve from random forest feature selection based on species-level microbial profiles. The model identified eight variables that minimized the prediction error, as indicated by the red vertical line.

####
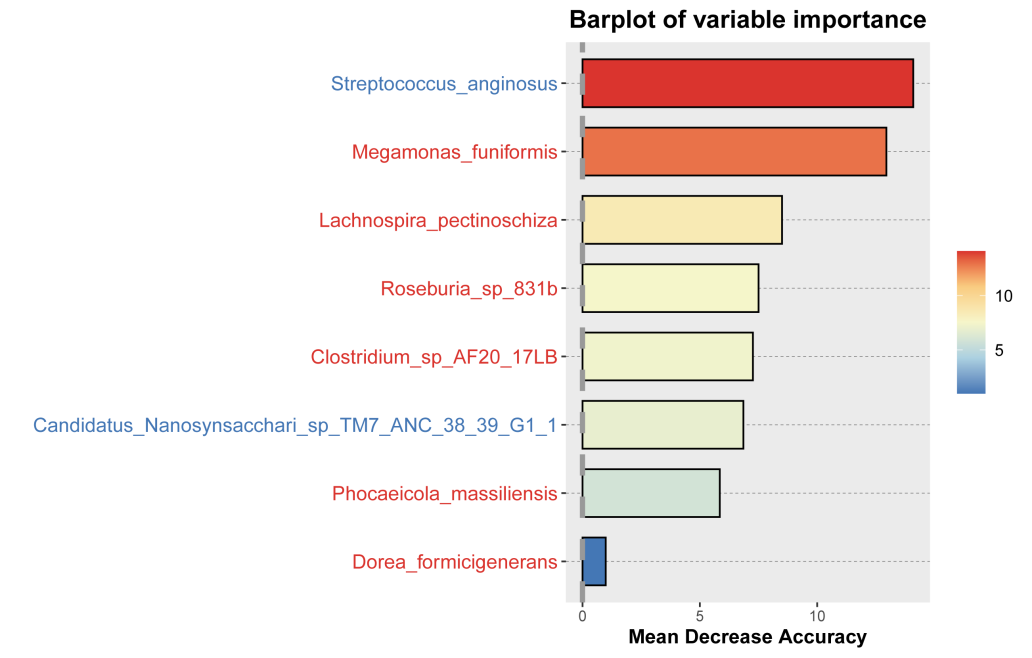


#### **Supplementary Figure 3.** Barplot of variable importance based on mean decrease in accuracy in the random forest model. Streptococcus anginosus, Megamonas funiformis, and Lachnospira pectinoschiza were the top contributors, suggesting their potential role as microbial biomarkers for AP classification.

## Supplementary Tables

**Supplementary Table 1 Antibodies used for Western blot analysis**

| **Target** | **Host** | **Catalogue No.** | **Manufacturer** | **Dilution** |
| --- | --- | --- | --- | --- |
| SRGN | Rabbit | 12985 | Cell Signaling Technology | 1:1000 |
| FTL | Mouse | sc-74513 | Santa Cruz Biotechnology | 1:1000 |
| F13A1 | Rabbit | sc-365423 | Santa Cruz Biotechnology | 1:800 |
| CEBPD | Rabbit | 12913 | Cell Signaling Technology | 1:1000 |
| RGS1 | Rabbit | sc-365272 | Santa Cruz Biotechnology | 1:500 |
| LGALS1 | Rabbit | 12935 | Cell Signaling Technology | 1:1000 |
| FN1 | Rabbit | sc-377433 | Santa Cruz Biotechnology | 1:800 |
| EGR1 | Rabbit | 4153 | Cell Signaling Technology | 1:1000 |
| GAPDH | Mouse | sc-47724 | Santa Cruz Biotechnology | 1:2000 |

**
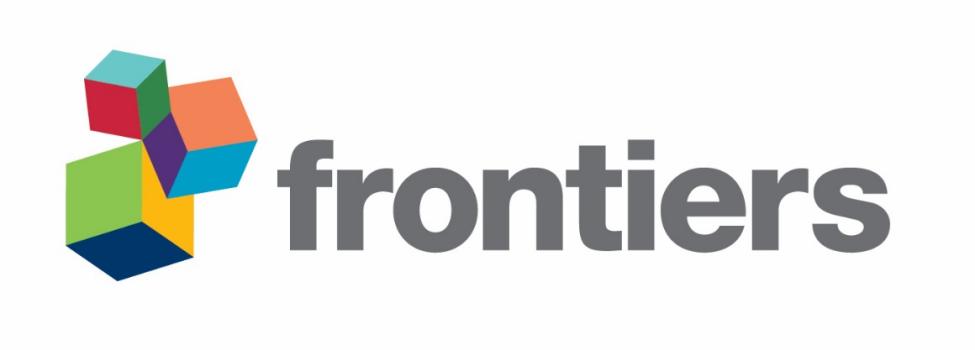
**
